# A small natural molecule CADPE kills residual colorectal cancer cells by inhibiting key transcription factors and translation initiation factors

**DOI:** 10.1038/s41419-020-03191-5

**Published:** 2020-11-15

**Authors:** Guo-Wan Zheng, Ming-Min Tang, Chen-Yan Shu, Wen-Xiu Xin, Yan-Hua Zhang, Bin-Bin Chi, Mu-Ran Shi, Xing Guo, Zhi-Zhen Zhang, Xiao-Yuan Lian

**Affiliations:** 1 College of Pharmaceutical Sciences, Zhejiang University, 310058 Hangzhou, Zhejiang China; 2 Department of Pharmacy, Zhejiang Cancer Hospital, 310022 Hangzhou, Zhejiang China; 3 Ocean College, Zhoushan Campus, Zhejiang University, 316021 Zhoushan, Zhejiang China

**Keywords:** Drug discovery, Drug development

## Abstract

Residual disease is the major cause for colorectal cancer (CRC) relapse. Herein, we explore whether and how a natural molecule CADPE killed heterogenic populations in a panel of CRC cell lines with KRAS/BRAF mutations that are natively resistant to EGFR- or VEGFR-targeted therapy, without sparing persistent cells, a reservoir of the disease relapse. Results showed that CADPE killed the tumor bulk and residual cells in the panel of CRC cell lines, rapidly inactivated c-Myc, STAT3, and NF-κB, and then decreased the protein levels of key signaling molecules for CRC, such as β-catenin, Notch1, and the nodes of mTOR pathways; eukaryotic translation initiation factors (eIF4F); anti-apoptotic proteins (Bcl-xl, Mcl-1, and survivin); and stemness-supporting molecules (CD133, Bim-1, and VEGF). In terms of mechanism of action, concurrent downregulation of Mcl-1, Bcl-xl, and survivin was necessary for CADPE to kill CRC bulk cells, while additional depletion of CD133 and VEGF proteins was required for killing the residual CRC cells. Moreover, the disabled c-Myc, STAT3, NF-κB, and eIF4F were associated with the broadly decreased levels of anti-apoptosis proteins and pro-stemness proteins. Consistently, CADPE suppressed CRC tumor growth associated with robust apoptosis and depleted levels of c-Myc, STAT3, NF-κB, eIF4F, anti-apoptotic proteins, and pro-stemness proteins. Our findings showed the promise of CADPE for treating CRC and suggested a rational polytherapy that disables c-Myc, STAT3, NF-κB, and eIF4F for killing CRC residual disease.

## Introduction

Colorectal cancer (CRC) is one of the most commonly diagnosed and lethal cancers^1^. Currently, chemotherapy is the standard of care for metastatic CRC. Targeted agents against epidermal growth factor receptor (EGFR) and vascular endothelial growth factor receptor (VEGFR) and the multi-tyrosine kinase inhibitor regorafenib are also approved for the treatment of advanced CRC^2,3^. However, in addition to severe side effects of chemotherapy, drug resistance eventually induces relapse from persisting cancer cells also termed residual disease^4^, due to dynamic and adaptive activation of oncogenic signaling pathways that bypass treatment pressure^5–7^. It is widely accepted that the residual disease is formed by cancer stem-like cells (CSCs) originally existed in tumor and transformed from differentiated cancer cells through the epithelial–mesenchymal transition (EMT) during the period of treatment^4^. Moreover, targeted drugs are limited by innate resistance through diverse mechanisms, such as a compensatory response of activated parallel pathways and autoactivated downstream effectors, such as Wnt/β-catenin, MAPK, and mTOR^8,9^. Thus, co-targeting of multiple nodes in one pathway or many pathways has been proposed^10,11^. Furthermore, multiple transcription factors (TFs), including c-Myc^12^, STAT3 (ref. ^13^), NF-κB^14^, and Notch^15^ are aberrantly activated in CRC cells and their transcriptional products support CRC tumorigenesis and progression. These hyperactivated TFs also promote resistance to chemotherapy and the targeted therapies largely through supporting original CSCs and promoting EMT^16–24^. Extensive efforts have been made to target multiple nodes and oncogenic TFs, but clinical success has not been achieved.

Accumulating evidences indicate that almost all of the major oncogenic signaling pathways converge on mRNAs translation to rewire the translational machinery and produce various oncogenic proteins, leading to tumorigenesis, cancer progression, and drug resistance^25^. Eukaryotic translation initiation factors 4E (eIF4E), eIF4G, and eIF4A have been shown to play crucial roles on oncogenic mRNAs translation^23,26^. First, Cap-dependent translation initiation requires eIF4A, eIF4E, and eIF4G to form the initiation translation complex^25,27^. Second, eIF4A and eIF4G also promote Cap-independent translations^28,29^. Many oncoproteins can be synthesized through either Cap-dependent or IRES-dependent translation^23^, including the signaling molecules and pro-tumor proteins that support CSCs and promote EMT^22,23,27,28^. Clearly, the deregulated translation provides promising targets that may overcome intratumor heterogeneity and selectively kill cancer cells^23,25^.

Caffeic acid 3,4-dihydroxyphenethylester (CADPE) is an anticancer natural product isolated from a water extract of the traditional Chinese medicine called Zhongjiefeng, the dried whole plant of *Sarcandra glabra* (Thunb) Nakai (Chloranthaceae). A Chinese patent medicine Zhongjiefeng injection made from the water extract of Zhongjiefeng is used for the treatment of gastric cancer, colon cancer, pancreatic cancer, liver cancer, and leukemia^30^. Our previous study showed that CADPE had broad-spectrum in vitro antitumor activity in 59 human cancer cell lines and in vivo antitumor effect in hepatoma H22 and sarcoma S180 tumor-bearing mice^31^. In this study, we explored the hypothesis that CADPE may kill residual CRC cells by inhibiting key TFs and translation initiation factors.

## Methods and materials

### Chemical agents and cell lines

CADPE (>98%) was synthesized by the authors^31^ and dissolved in DMSO for in vitro assay or in hydroxypropyl-β-cyclodextrin for in vivo experiments. Inhibitors ABT737 (737 for Bcl-xl), A-1210477 (477 for Mcl-1), YM155 (155 for survivin), Bay 11-7085 (Bay for NF-κB), ruxolitinib (Rux for STAT3), 10058-F4 (F4 for c-Myc), and 4EGI-1 (4EGI for Cap-translation) and positive control drug regorafenib (Rego) were purchased from the MedChemexpress Co., Ltd. All CRC cells were obtained from the China Type Culture Collection (Shanghai) and normal colon fibroblast CCD-18Co cells from the Shanghai Bogoo Biotechnology Co., Ltd. HCT-8, HCT-15, and CT26.WT cells were cultured in RPMI-1640 (Gibco), HCT-116 and HT-29 cells in McCOY′5A (Gibco), SW620 cells in Leiboviz′s L15 (Gibco), and CCD-18Co cells in DMEM (Gibco), supplemented with 2 mM l-glutamine. All cells were grown in medium with 10% fetal bovine serum (FBS), penicillin (20 U/mL), and streptomycin (20 μg/mL). Cells were authenticated by STR profiling and routinely screened for the presence of *Mycoplasma* by EZ-PCR Mycoplasma test Kit (Biological Industries).

### Cell viability assay

Cells were seeded in 96-well plates at a density that generated continual linear growth and treated with tested agents for 72 h. Cell viability was measured by the sulforhodamine B assay in triplicate.

### Analysis of apoptosis and mitochondrial membrane potential (MMP)

According to the experimental purposes, cells were treated with the tested agents for 48 and 72 h and then double stained by Annexin V-FITC/PI using an Annexin V apoptosis detection kit (Multi Sciences Biotech). The apoptosis rate was analyzed by flow cytometry with a flow cytometer and the FlowJo software. MMP was determined by a fluorescent probe JC-1 (Beyotime Biotechnology) as previously described^32^. The ΔΨm was indicated by the fluorescent ratio of red/green.

### Western blotting and quantitative real-time polymerase chain reaction (qRT-PCR)

Whole-cell lysates from cells were prepared in RIPA lysis buffer containing protease inhibitor cocktail and phosphatase inhibitor (Roche). The protein lysates were denatured and used for western blotting using standard method^33^. The primary antibodies and horseradish peroxidase secondary antibodies used are shown in Table S1 (Supplementary data).

Total RNA was extracted from cells using Trizol reagent (Invitrogen). First-strand cDNA was synthesized from 500 ng of total RNA using PrimeScript™ RT reagent Kit with gDNA Eraser (Takara). The cDNA was used as the template for real-time quantity PCR (Bio-Rad CFX96). The sequences of the primers used in this study are listed in Table S2. After the standard Bio-Rad cycling program, the melting curve of amplification products was analyzed, and qRT-PCR data were collected as Ct value. The relative expression level of gene was calculated by the 2−ΔΔCt method.

### Immunofluorescence

Immunofluorescence staining was performed using standard methods. Briefly, after the treatment, cells were fixed and permeabilized with 4% paraformaldehyde for 10 min, followed by washing twice using phosphate-buffered saline (PBS) with 0.1% Tween 20 and then blocking with 3% bovine serum albumin in PBS. All the specimens were stained with a primary antibody and a labeled secondary Alexa Fluor 488 or 594 antibody (Jackson ImmunoResearch). Nuclei were stained using 4′,6-diamidino-2-phenylindole. The mitochondria were labeled with Mito Tracker® Red (100 nM) (Invitrogen) in the medium without FBS for 20 min at 37 °C in a 5% CO_2_ incubator. Fluorescent images were obtained with a Nikon ECLIPSE 50i microscope.

### Sphere culture and mammosphere formation assay

HCT-15 or HCT-116 cells were collected and suspended in serum-free DMEM/F12 supplemented with B27, rhEGF, and rhbFGF, which was named as growth factor defined serum-free medium. The cells were subsequently cultured in ultra-low attachment six-well plates at a density of 2000 cells/mL. To passage the sphere cells, spheres were collected by gentle centrifugation, then dissociated with 0.05% trypsin, 0.5 mM EDTA and mechanically disrupted by a pipette. The resulting single cells were centrifuged and then re-suspended in growth factor defined serum-free medium to re-form spheres. The spheres should be passaged every 5–8 days before they reached a diameter of 100 μm. All the sphere cells used in this study were within 20 generations.

For mammosphere formation assay, after the treatment, cells were collected and transferred to ultra-low attachment 96-well plates at a density of 2000 cells/mL in growth factor defined serum-free medium for culturing. Seven days later, the number of spheres were counted, and cell morphology was observed under a microscope.

### Animal experiments

All animal procedures were approved by the Institutional Animal Care and Use Committee of Zhejiang University. All mice were purchased from the Shanghai SLAC laboratory Animal Co., Ltd. They were housed in a specific pathogen-free facility with four mice in one cage and had free access to standard food and water under a 12-h light/12-h dark cycle at a constant temperature of 23 ± 2 °C and 55% humidity. The experiments were performed after the mice were allowed to acclimate for 1 week. Investigators were blinded to allocation during in vivo experiments and outcome assessments.

For the SW620 xenograft mice model, SW620 cells (5 × 10^6^) suspended in 100 μL of FBS-free medium were injected into the right shoulder of the six-week-old BALB/c nude mice (male, 16–18 g). Caliper measurements begun when tumors became visible and tumor volume was calculated using the following formula: tumor volume = (*D* × *d*^2^)/2, where *D* and *d* refer to the long and short tumor diameter, respectively. When the tumor volume reached to 80–120 mm^3^, mice were randomized to following groups (saline, vehicle, CADPE, *n* = 5–6). CADPE (25 mg/kg, i.p.), saline, or vehicle was administrated once a day until the tumor volume in the saline controls reached round 1400 mm^3^ and then animals were euthanized to collect tumors. Another set of the xenografts were used for intratumor injection. When tumor volume reached to around 1200 mm^3^, xenografts were assigned to receive injection of vehicle or CADPE (*n* = 3) for one, two, or three times through evenly distributed five injection sites (1 μL of 50 μM CADPE or vehicle for each site), and the interval between the injection time was 72 h. At 24 h after the last drug administration, animals were euthanized to collect tumors. All collected tumors were fixed in formalin and then embedded in paraffin for hematoxylin and eosin (HE) staining and immunohistochemistry (IHC).

For the CT26.WT tumorigenicity experiment, cells were seeded in 6 cm dishes in RPMI-1640 medium with 10% FBS at a density of 4.5 × 10^5^ cells per dish for 24 h. After the treatment of CADPE (75 μM) or Rego (20 μM) for 48 h, the residual cells were harvested and counted with trypan blue. The cells (2 × 10^5^) in a volume of 100 μL of FBS-free medium from the CADPE or Rego group were subcutaneously injected into the right shoulder of the six-week-old BALB/c mice (male, 16–18 g). After 14 days, animals were euthanized, and tumors were collected, weighted, and photographed.

### Immunohistochemistry

The embedded tumor tissue was sectioned into 5 μm slices. After heating and antigen retrieval (incubating the slices at 98 °C in Tris-EDTA buffer for 5 min), tissue sections were subjected to IHC analysis according to standard protocol^34^. Primary antibodies (Table S1) and secondary goat anti-mouse or anti-rabbit antibody conjugated with horseradish peroxidase (PV-9000 Kit, ZSGB-BIO) were used. The negative controls were made by omitting the primary antibodies for each staining. Target proteins were visualized with diaminobenzidine and images were obtained with a Nikon ECLIPSE 50i microscope.

### Statistical analysis

All data are displayed as the mean ± SD from triplicated independent experiments. Numbers (*n*) for tested groups are stated in the figure legends. GraphPad Prism 5.0 was used for statistical analysis. Comparisons between two groups were carried out with two-tailed Student′s *t*-test. Variances among multiple groups were analyzed with one-way ANOVA followed by Tukey**’**s test. The *p* value < 0.05 was considered to indicate the statistical significance.

## Results

### CADPE induces apoptosis in CRC cells

**Fig. 1:**
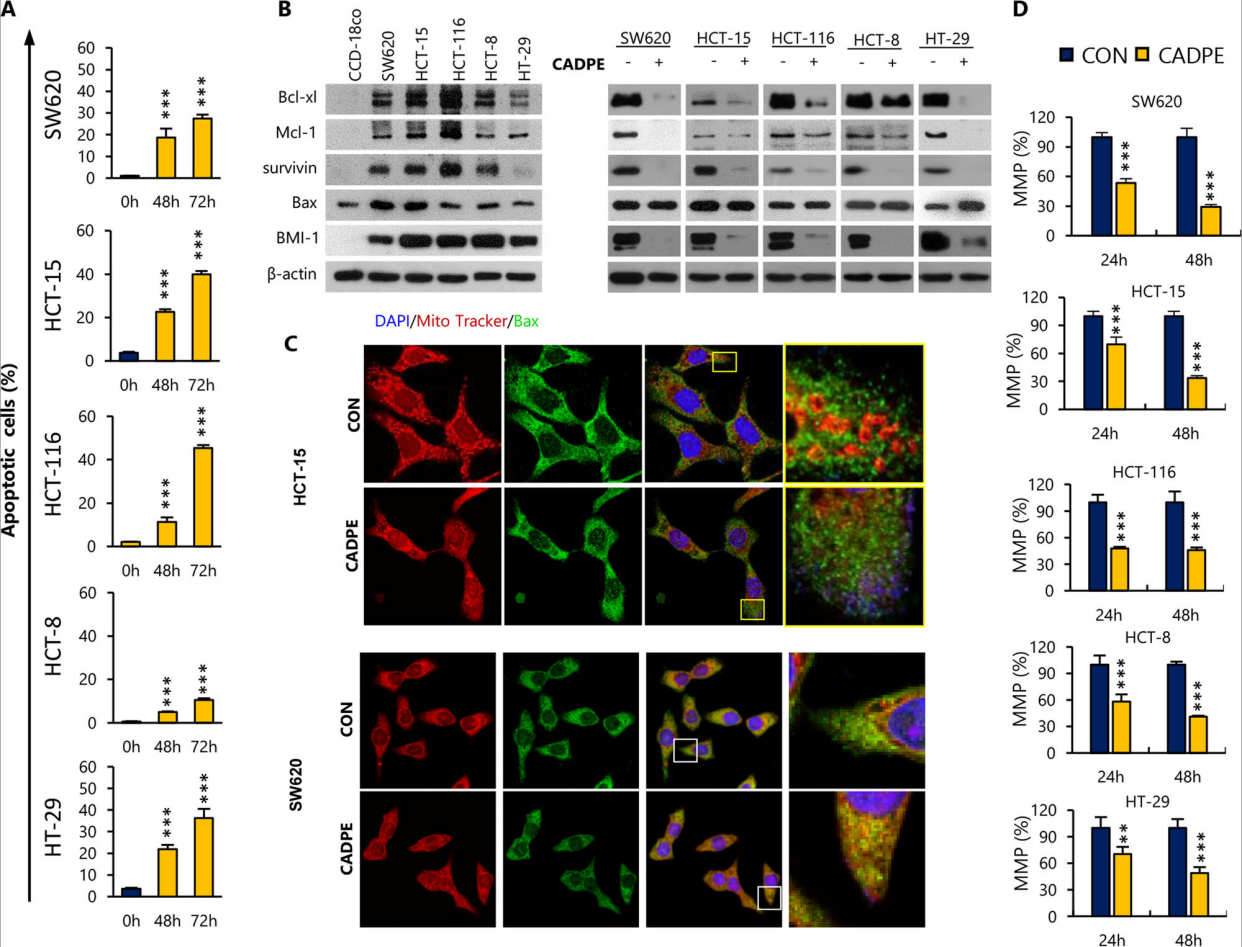
CADPE induces apoptosis in CRC cells. **a** Apoptosis rates of CRC cells were measured by flow cytometry after treating with CADPE (25 μM) for 48 and 72 h (*n* = 3). **b** Expression levels of anti-apoptotic proteins in normal colon CCD-18Co and CRC cells and effect of CADPE (25 μM) on the expression levels of above anti-apoptotic proteins in CRC cells after the treatment of 48 h through immunoblot analysis. **c** HCT-15 and SW620 cells treated with CADPE (12.5 μM) for 24 h were subjected to immunocytological analysis. Bar: 30 μm. **d** Mitochondrial membrane potential (MMP) in CRC cells was altered after the treatment of CADPE (12.5 μM) for 24 and 48 h (*n* = 4). Data are presented as the mean ± SD, ***p < 0.001 (vs. CON) by one-way ANOVA followed by Tukey’s test.

The results (Fig. 1a and Fig. S1a) showed that CADPE induced robust apoptosis in the tested five cell lines except in HCT-8 cells, which tended to be senescent (Fig. S1b). The protein levels of Bcl-xl, Mcl-1, and survivin, which have been reported to protect cancer bulk cells and residual disease against treatment^35^, expressed higher in CRC cells than in normal CCD-18Co cells and CADPE decreased these pro-survival proteins with an exception for Bcl-xl, which was mildly decreased in HCT-8 (Fig. 1b). CADPE did not upregulate the levels of Bax (Fig. 1b) and other pro-apoptosis protein (data not shown). However, CADPE increased Bax translocation into mitochondria (Fig. 1c) with a decreased MMP (Fig. 1d). These data indicated that CADPE induced Bax mitochondrial translocation by decreasing the pro-survival protein levels^36^, resulting in the apoptosis. Importantly, CADPE had little effect on cell viability of normal CCD-18Co cells and the cytotoxicity concentration of CADPE against CCD-18Co cells was much higher than that of the positive control drug regorafenib (Rego)^37^ (Fig. S2).

### CADPE induces apoptosis through co-targeting of Bcl-xl, Mcl-1, and survivin

**Fig. 2:**
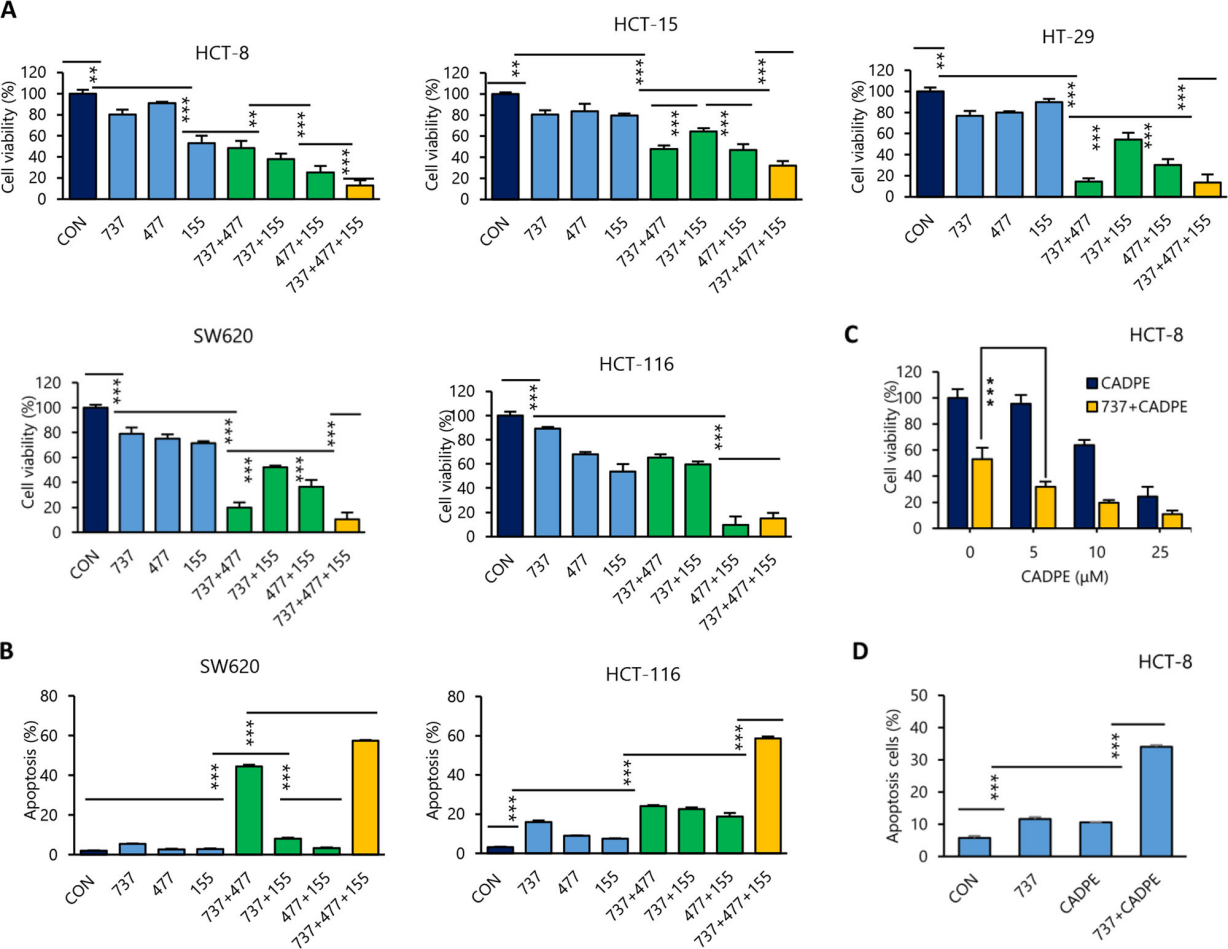
Simultaneous inhibition of Bcl-xl, Mcl-1, and survivin is required for CADPE to induce heterogenetic CRC cells to apoptosis. **a**, **b** Cell viability and apoptosis of CRC cells treated with Bcl-xl inhibitor ABT737 (737, 0.5 and 3 μM), Mcl-1 inhibitor A-1210477 (477, 2 and 6 μM), survivin inhibitor YM155 (155, 3 and 1 μM), and a combination of two inhibitors or three inhibitors for 72 and 48 h, respectively (*n* = 5 for cell viability, *n* = 3 for apoptosis). **c** Cell viability of HCT-8 cells treated with CADPE (5, 10, 25 μM) and its combinations with 737 (4 μM) for 72 h (*n* = 5). **d** Apoptosis induced by 48 h treatment of CADPE (12.5 μM), 737 (3 μM), and their combination in HCT-8 cells (*n* = 3). Data are presented as the mean ± SD, **p < 0.01, ***p < 0.001 (vs. corresponding group) by one-way ANOVA followed by Tukey’s test.

We tested whether the concurrent downregulation of three anti-apoptotic proteins (Bcl-xl, Mcl-1, and survivin) is required for the CADPE-induced apoptosis. The three proteins were inhibited by using their chemical inhibitors 737 for Bcl-xl, 477 for Mcl-1, and 155 for survivin. As shown in Fig. 2a, each inhibitor alone slightly decreased cell viability except 155 in HCT-8 and HCT-116 cells. A combination of any two inhibitors produced synergistic effects with varying degrees in cell context with potent effects for the combination of 737 and 477 in HT-29 and SW620 cells and the combination of 477 and 155 in HCT-116 cells. Particularly, the combination of three inhibitors significantly reduced cell viability in all tested cells. Consistent results were also obtained from apoptosis analyses in HCT-116 and SW620 cells (Fig. 2b and Fig. S3a). Furthermore, a co-treatment of CADPE and Bcl-xl inhibitor 737 potently decreased cell viability (Fig. 2c) and induced robust apoptosis (Fig. 2d and Fig. S3b) in HCT-8. Together, these data indicated that concurrent inhibition of Bcl-xl, Mcl-1, and survivin was necessary for inducing heterogenic CRC cells into robust apoptosis and contributive to the proapoptotic effect of CADPE.

### CADPE kills residual CRC cells through mechanisms beyond targeting multi-kinases

**Fig. 3:**
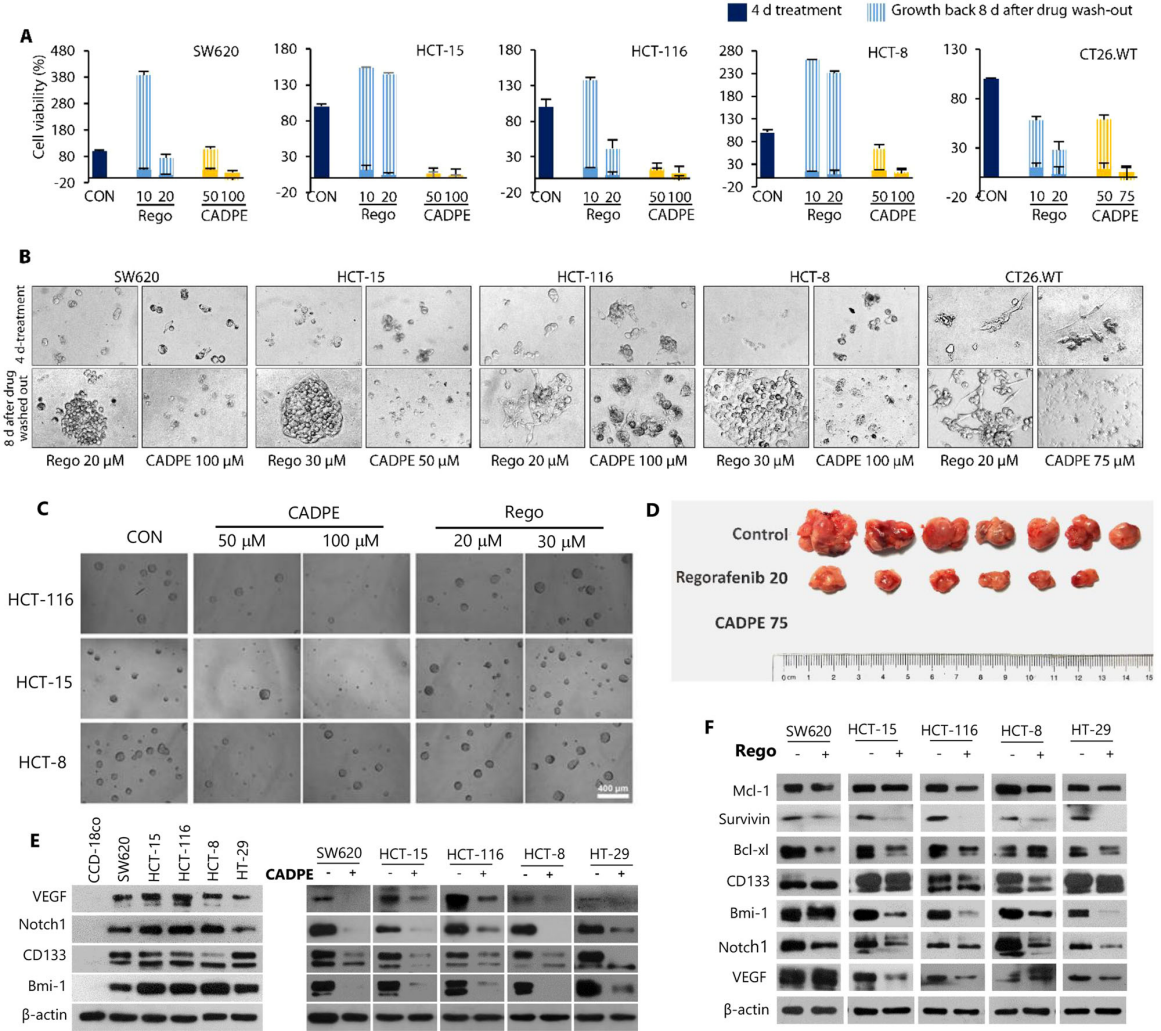
CADPE kills heterogenic cancer cells in different populations of CRC cells. **a**, **b** Cell viability and representative images of SW620, HCT-15, HCT-116, HCT-8, and CT26.WT cells. Cells were incubated with regorafenib (Rego) or CADPE with different concentrations for 4 days and allowed for growth of additional 8 days after removing drugs (*n* = 4). **c** HCT-116, HCT-15, and HCT-8 cells were dissociated to single cells and allowed to form spheres in cancer stem cell medium for 7 days after the treatment of 48 h with CADPE (50, 100 μM) or Rego (20, 30 μM). **d** CT26.WT cells pretreated with Rego (20 mg/kg) or CADPE (75 mg/kg) for 48 h were subcutaneously injected into BALB/c mice. After 14 days, tumors were harvested and measured with a Vernier caliper (*n* = 7). **e** Protein levels in CCD-18Co and CRC cells and CRC cells treated with CADPE (25 μM) for 48 h through immunoblot analysis. **f** Protein levels in CRC cells treated with Rego (15 μM) for 48 h through immunoblot analysis.

Currently, anticancer therapies always cause EMT through which cancer cells obtain stem cell-like properties and remain in a dormant state as a residual disease responsible for tumor relapse^19,38,39^. Therefore, we first tested whether CADPE could kill CRC residual cells. The results (Fig. 3a, b) showed that both CADPE and Rego remarkably decreased cell viability with few remaining cells (residual cells). CADPE-treated residual cells were destroyed and unable to resume growth. However, Rego-treated residual cells continued to grow during the period of additional culture, although Rego induced more apoptosis than CADPE at the tested doses in the most CRC cell lines (Fig. S4). This unique effect of CADPE was further confirmed in a in vivo study. CADPE- or Rego-treated residual CT26.WT cells were implanted into mice and allowed to grow for 14 days. In line with the cellular results, the Rego-treated residual cells grew to tumor, whereas the CADPE-treated residual cells lost their tumorigenicity (Fig. 3d). These results suggested that CADPE was capable to prevent the recurrence CRC cells and the formation of tumors in the in vivo assay. However, Rego had no such effect. To further support above results, CADPE and Rego were evaluated for their activities to kill heterogeneous CRC cells without leaving the cells with stem cell-like properties in HCT-8, HCT-15, and HCT-116 cells by using mammosphere formation assay, a technique used to measure the sphere forming ability of cancer stem cells (CSCs)^17^. As expected, CADPE suppressed the sphere formation of the parental cells from different CRC cell lines, whereas regorafenib had no obvious inhibitory effect on the sphere formation of the parental cells (Fig. 3c).

Next, CADPE and Rego were tested for their ability to produce a direct effect on CSCs, which have been considered as an important reason for tumor recurrence^40^. Enriched CRC stem-like cells (CRCSCs) were initially isolated from the parental HCT-8, HCT-15, and HCT-116 cells, by forming tumorspheres in growth factor defined serum-free medium. To confirm the stemness of the isolated CRCSCs, the expression profiles were compared between the parental CRC cells and CRCSCs, using qRT-PCR and western blot assays. As expected, the upregulated mRNA levels (Fig. S5a) of putative stemness markers of CD44 (1.96-fold), CD133 (4.23-fold), and Notch1 (8.44-fold) were observed in the HCT-116 CSCs. A consistent result was also obtained from the analysis of the protein levels of these stemness markers (Fig. S5b). Both CADPE and Rego significantly inhibited the proliferation of the HCT-8, HCT-15, and HCT-116 CSCs (Fig. S5c) and decreased the formation of mammospheres in HCT-15 and HCT-116 CSCs (Fig. S6a, b). Furthermore, CADPE caused disintegration of the preformed tumorspheres of HCT-15 and HCT-116 CSCs with obvious apoptotic characteristics (Fig. S6c, d). CADPE also downregulated stemness makers (CD44, CD133, Bmi-1, and Notch1), anti-apoptotic proteins (Bcl-xl, Mcl-1, and survivin), and key oncogenic transcriptional factors (p-STAT3, STAT3, and c-Myc) (Fig. S7). However, Rego did not disintegrate the preformed tumorspheres of HCT-15 and HCT-116 CSCs and only reduced the size of tumorspheres (Fig. S6c), suggesting that the residual cells surviving from the Rego treatment were not the original CSCs, but transformed from more differentiated cancer cells through EMT instead.

The different mechanisms between CADPE and Rego were used to identify potential molecules linked to the residual disease. First, pro-stemness proteins including VEGF^41^ and stemness biomarkers (Notch1, Bmi-1, and CD133) that regulate and support the ability of colorectal CSCs to self-renew^17^ were identified to overexpress in the different CRC cell lines (Fig. 3e). CADPE potently downregulated the levels of all these pro-stemness proteins (Fig. 3e). Rego exhibited a weaker efficiency on Bcl-xl, Noch1, Bmi-1 than CADPE and decreased VEGF in some CRC cell lines with no obvious effect on the CD133 levels in all tested cell lines (Fig. 3f). Given the key roles of Mcl-1 and Bcl-xl against CRC apoptosis resistance and autocrine VEGF signaling and CD133 in protecting cancer cells against drug treatment, in promoting EMT, and in supporting stem cell phenotype^41^, one could believe that the partially maintained pro-survival molecules (Mcl-1, Bcl-xl, VEGF, and CD133) may protect the residual cells against Rego.

### CADPE abolishes the key nodes of major oncogenic signaling pathways

**Fig. 4:**
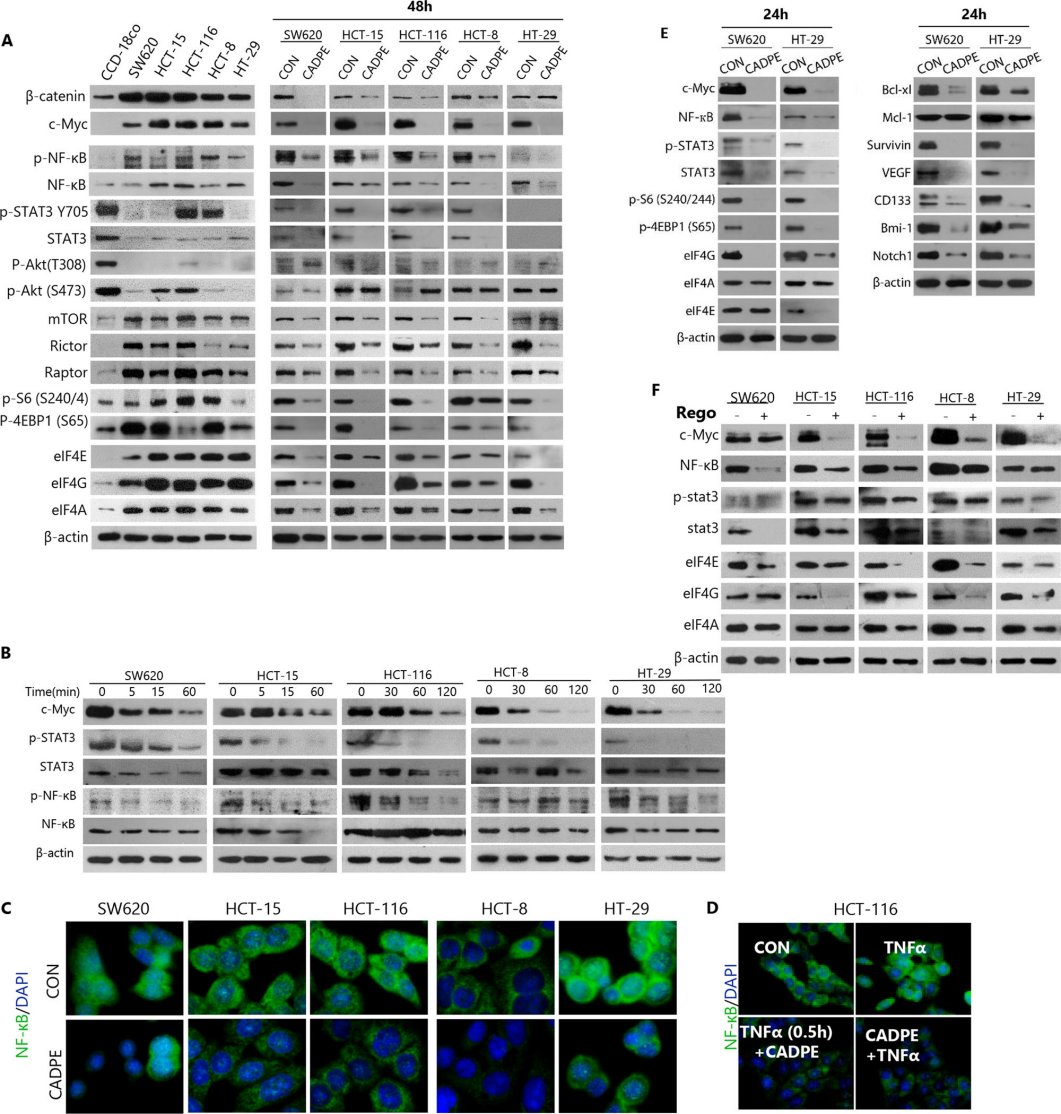
CADPE reverses signal and molecular characteristics of CRC cells. **a** Protein levels of the key transcription factors, master regulator in mTOR signaling and its downstream targets, translation initiation factors, anti-apoptotic proteins, and cancer cell stemness-related proteins in CCD-18Co and CRC cells and CADPE-treated CRC cells with a concentration of 25 μM for 48 h through immunoblot analysis. **b** Protein levels of c-Myc, p-STAT3, STAT3, p-NF-κB, and NF-κB in CRC cells treated with CADPE (25 μM) at the indicated time points. **c** Immunocytological analysis of NF-κB in CRC cells treated with 10% FBS and CADPE (12.5 μM) for 6 h after serum starvation for 18 h. Bar: 20 μm. **d** Immunocytological analysis of NF-κB in HCT-116 cells treated with TNF*α* (50 ng/mL) combined with CADPE (12.5 μM) for 3 h, or pretreated with TNFα (50 ng/mL) for 30 min and then treated with CADPE for 3 h, after serum starvation for 18 h. Bar: 20 μm. **e** Protein levels in CRC cells treated with CADPE (25 μM) for 24 h through immunoblot analysis. **f** Protein levels in CRC cells treated with Rego (15 μM) for 24 h through immunoblot analysis.

In order to study how CADPE decreased those oncogenic proteins that protect CRC cells from apoptosis and support CRC residual disease. The effects of CADPE on both transcriptional and translational key regulators in CRC cells were investigated, including oncogenic signaling from divergent pathways β-catenin, c-Myc, NF-κB, and STAT3 (refs. ^4,42^) and key nodes of translation system mTOR pathways (mTOR, Raptor, Rictor, p-S6, p-4EBP), eIF4F (eIF4E, eIF4A, and eIF4G), all of which drive CRC and are associated to residual disease in solid cancers. Cancer cells quickly adapt to that drug, so as to maintain the signal flux through those networks required for tumor maintenance and growth^25,43^. Strikingly, all the tested CRC cell lines had highly activated signaling of oncogenic transcription and translation reprograming as evidenced by increased levels of β-catenin, c-Myc, NF-κB, and p-NF-κB, the signaling nodes (mTOR, Raptor, Rictor, p-S6, p-4EBP), and eIF4F. A 48 h treatment of CADPE potently decreased the levels of these molecules. Importantly, c-Myc, p-STAT3, p-NF-κB, p-S6, eIF4G, and eIF4A were the molecules most downregulated by CADPE (Fig. 4a).

The results also showed that CADPE dramatically decreased the levels of c-Myc, p-STAT3, and p-NF-κB within 1 h (Fig. 4b), which maybe the potential molecules initially affected by CADPE. Immunocytochemistry assay showed that CADPE decreased nuclear entry of NF-κB at 6 h (Fig. 4c). To confirm the effect of CADPE on NF-κB signaling, the nuclear NF-κB levels were measured when CADPE and TNFα were adminstrated simutaneously (TNFα + CADPE) or CADPE was added 0.5 h later (TNFα 0.5 h + CADPE) in HCT-116. CADPE completely counteracted TNFα from enhancing NF-κB levels and its nuclear entry, even to the levels much lower than baseline, regardless of the order of addition (Fig. 4d). However, eIF4F and the other pro-tumorigenic proteins were not decreased at the early time points and even at 24 h (data not shown), and CADPE did not affect the levels of eIF4A and Mcl-1 (Fig. 4e), which is consistent with a previous report that selective inhibition of eIF4A decreased Mcl-1 level in leukemia cells^26^. But, CADPE downregulated most of the tested pro-tumorigenic proteins (Fig. 4e). Collectively, the observations indicated that CADPE rapidly inhibited c-Myc, p-NF-κB, and p-STAT3, followed by broad downregulation of eIF4F, signaling nodes, and pro-survival molecules, suggesting that CADPE can downregulate tumorigenic proteins including ones supporting residual cells at both transcription and translation levels. Of note, p-Akt (T308) and p-Akt (S473) levels decreased in all tested CRC cells, when compared to normal CCD-18Co cells, and CADPE seemingly did not affect the levels of the two proteins (Fig. 4a). In line with this observation, the downstream mTORC1 activity has been reported to be transcriptionally upregulated by ERK in KRAS mutant CRC cells, thereby causing resistance to PI3K and Akt inhibitors^18^.

To further identify the potential molecules that fuel CRC residual disease, the effect of Rego on the levels of those oncogenic signaling molecules was also evaluated. Rego also dramatically downregulated the levels of c-Myc in nearly all tested cell lines, but failed to simultaneously downregulate NF-κB, STAT3, p-STAT3, and eIF4F in the cell lines (Fig. 4f). Together with the inhibitory effects of CADPE on these key TFs and eIF4F, these data suggested that the preserved key TFs and eIF4F may work together to produce Mcl-1, Bcl-xl, CD133, and VEGF and thereby support residual disease after Rego treatment.

**Fig. 5:**
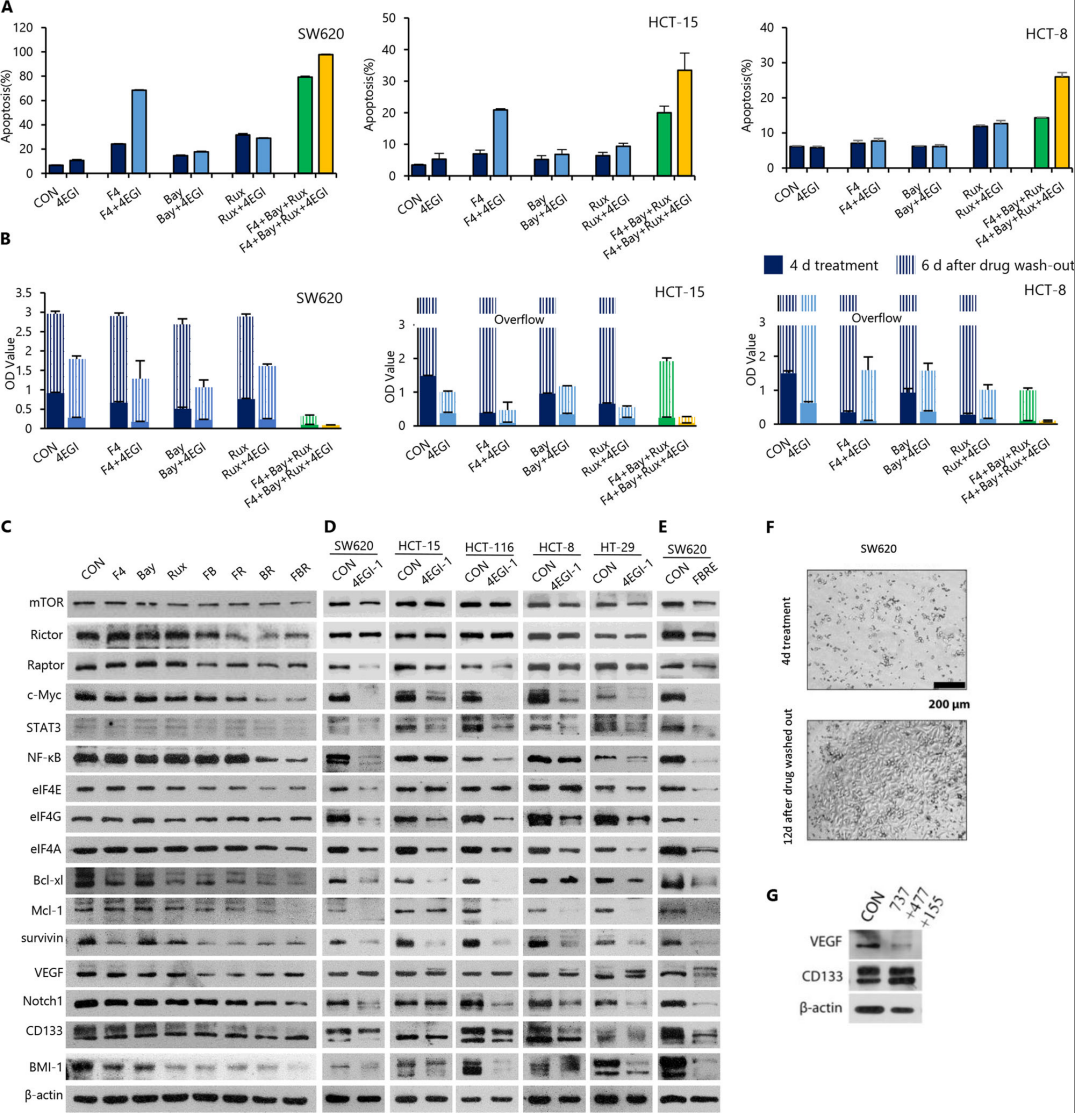
Dual inhibition of multiple oncogenic transcription factors and protein translation can mimic the effects of CADPE. **a** c-Myc inhibitor (10058-F4, F4, 30 μM), STAT3 inhibitor (Ruxolitinib, Rux, 20 μM), NF-κB inhibitor (Bay 11-7085, Bay, 20 μM), competitive eIF4E/eIF4G interaction inhibitor (4EGI-1, 4EGI, 10 μM), or their random combinations induced apoptosis in SW620, HCT-15, and HCT-8 cells (*n* = 3). **b** Optical density (OD) values indicated an increase in cells viability of SW620, HCT-15, and HCT-8 cells incubated with individual inhibitor, or combined inhibitors for 4 days and allowed for growth of additional 8 days after removing the treatments (*n* = 4). **c**–**e** Protein levels of the key transcription factors and master regulators in mTOR signaling and its downstream targets, translation initiation factors, anti-apoptotic proteins, and cancer stemness-related proteins in SW620 cells treated with c-Myc inhibitor F4, NF-κB inhibitor Bay, STAT3 inhibitor Rux, and their random combinations, or in SW620, HCT-15, HCT-116, HCT-8, and HT-29 cells treated with 4EGI (50 μM), or in SW620 cells treated with a combination of F4 (30 μM), Bay (20 μM), Rux (20 μM), and 4EGI (10 μM) for 48 h. **f**, **g** SW620 cells and protein levels of VEGF and CD133 after the treatment of a combination of Bcl-xl inhibitor ABT737 (737, 0.5 μM), Mcl-1 inhibitor A-1210477 (477, 2 μM), and survivin inhibitor YM155 (155, 3 μM) for 4 days and allowed for growth of additional 12 days after the treatment were removed. FB: a combination of F4 and Bay; FR: a combination of F4 and Rux; BR: a combination of Bay and Rux; FBR: a combination of F4, Bay, and Rux; FBRE: a combination of F4, Bay, Rux, and 4EGI. Data are presented as the mean ± SD.

To confirm the above proposal, the effects of different inhibitors on cell viability and apoptosis in CRC cell lines were evaluated, including F4 for c-Myc, Rux for STAT3, Bay for NF-κB, and 4EGI for eIF4E–eIF4G interaction. As predicted, a combination of three TFs inhibitors (F4, Rux, and Bay) induced robust apoptosis, which was further elevated when 4EGI was added in all tested cell lines (Fig. 5a and Fig. S8), at the doses that showed no or weak activity when used alone or combined 4EGI with one inhibitor of the TFs. Accordingly, the CRC cells treated with the combined four inhibitors for 4 days did not grow back in SW620 and HCT-8 cells or barely grew in HCT-15 cells after drug withdrawal (Fig. 5b). These findings demonstrated that concurrent inhibition of the three TFs and Cap-dependent translation could reproduce the activity of CADPE in inducing apoptosis and killing CRC cells. Therefore, dual inhibition of the three key TFs and eIF4s is crucial for CADPE to kill heterogenetic and residual CRC cells.

Importantly, this dual inhibition also reproduced the molecular effects of CADPE. First, only the combination of three TF inhibitors downregulated the key regulators of apoptosis, but did not affect the levels of eIF4A, eIF4G, VEGF, and CD133 in SW620 cells (Fig. 5c). Second, 4EGI decreased most of these oncoproteins in SW620 cells and fewer in other cell lines, but with no effects on the levels of CD133 and VEGF in all tested cell lines (Fig. 5d). However, dual inhibition of the Cap-dependent translation and the three TFs remarkably downregulated all 16 oncoproteins in SW620 cells (Fig. 5e). These observations further indicated that CD133 together with VEGF or each alone may be enough for protecting residual disease.

To verify this proposed role of CD133 and VEGF, we evaluated whether co-inhibition of Mcl-1, Bcl-xl, and survivin, which induced robust apoptosis (Fig. 2b), would affect the functional residual cells and the correlated status of CD133 and VEGF in SW620 cells. The co-inhibition of Mcl-1, Bcl-xl, and survivin by using their chemical inhibitors for 4 days killed the majority of SW620 cells with dramatically decreased VEGF but elevated CD133 levels (Fig. 5f, g). A large number of cancer cells grew back from the residual cells 12 days later after treatment withdrawal (Fig. 5f). Together with the early obtained results that Rego decreased VEGF in some CRC cell lines with no effect on the CD133 levels in all tested cell lines (Fig. 3f), our findings indicated that any of VEGF and CD133 might be able to support functional residual cancer cells, leading to CRCs recurrence, and that CADPE broadly downregulates oncoproteins including ones supporting residual disease through the inhibition of the three key TFs and the Cap-dependent translation.

### CADPE suppresses CRC tumor growth, induces apoptosis, and depletes tumorigenic signaling nodes and pro-tumor proteins in vivo

**Fig. 6:**
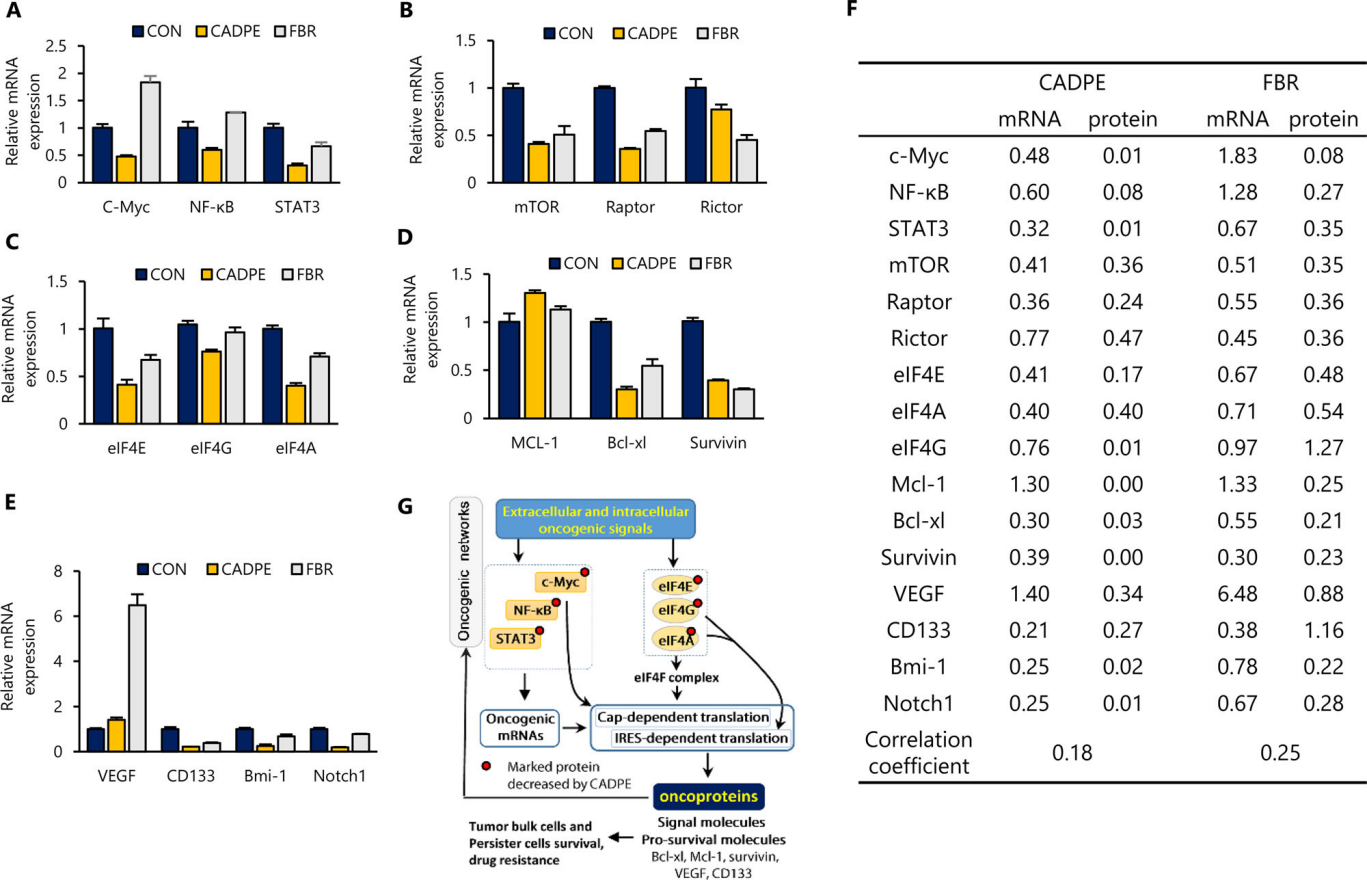
Effects of CADPE and the dual inhibition of the key transcription factors and protein translation on the mRNA levels of key regulators. **a**–**e** Relative mRNA levels of key transcription factors, master regulators in mTOR signaling and its downstream targets, translation initiation factors, anti-apoptotic proteins, and cancer stemness-related proteins in SW620 cells treated with CADPE (12.5 μM) or FBR for 24 h (*n* = 3). FBR: a combination of 10058-F4 (30 μM), Bay 11-7085 (20 μM), and Ruxolitinib (20 μM). **f** mRNA and protein levels relative to control after treated with CADPE (12.5 μM) or FBR, normalized to β-actin. The relative protein levels were calculated according to the following formula: (gray level of target protein band/gray level of β-actin from the treatment group)/(gray level of target protein band/gray level of β-actin in control group). **g** Schematic overview of mechanisms underlying anti-CRC effect of CADPE.

As shown in Fig. 6a–e, CADPE downregulated the expression of those genes targeted by FBR, including mTOR, Raptor, Rictor, eIF4E, eIF4A, CD133, Bcl-xl, and Survivin, and CADPE also decreased other genes that encoded eIF4G, Bmi-1, Notch1, c-Myc, NF-κB, and STAT3, likely because of the CADPE-downregulated protein levels of other TFs such as β-catenin and Notch1. However, the extent of the decreased mRNA levels induced by FBR or CADPE was much lower than that of their decreased protein levels, and many decreased pro-tumorigenic proteins did not show a corresponding decrease in the mRNA level (Fig. 6f). In contrast, the mRNA levels of VEGF and Mcl-1 were elevated by both FBR and CADPE (Fig. 6d, e). This disconnection between protein and mRNA abundances is consistent with the notion that both mRNA abundance and translational regulation affect protein levels^25,44,45^. Together, the data demonstrated that the co-inhibition of c-Myc, NF-κB, and STAT3 not only decreased oncogenic mRNA levels but also affected their translation, likely because of disabled c-Myc function that enhances translation^46^, and CADPE downregulated oncogenic proteins by suppressing both transcription and translation, but the impeded translation made a greater contribution (Fig. 6g).

**Fig. 7:**
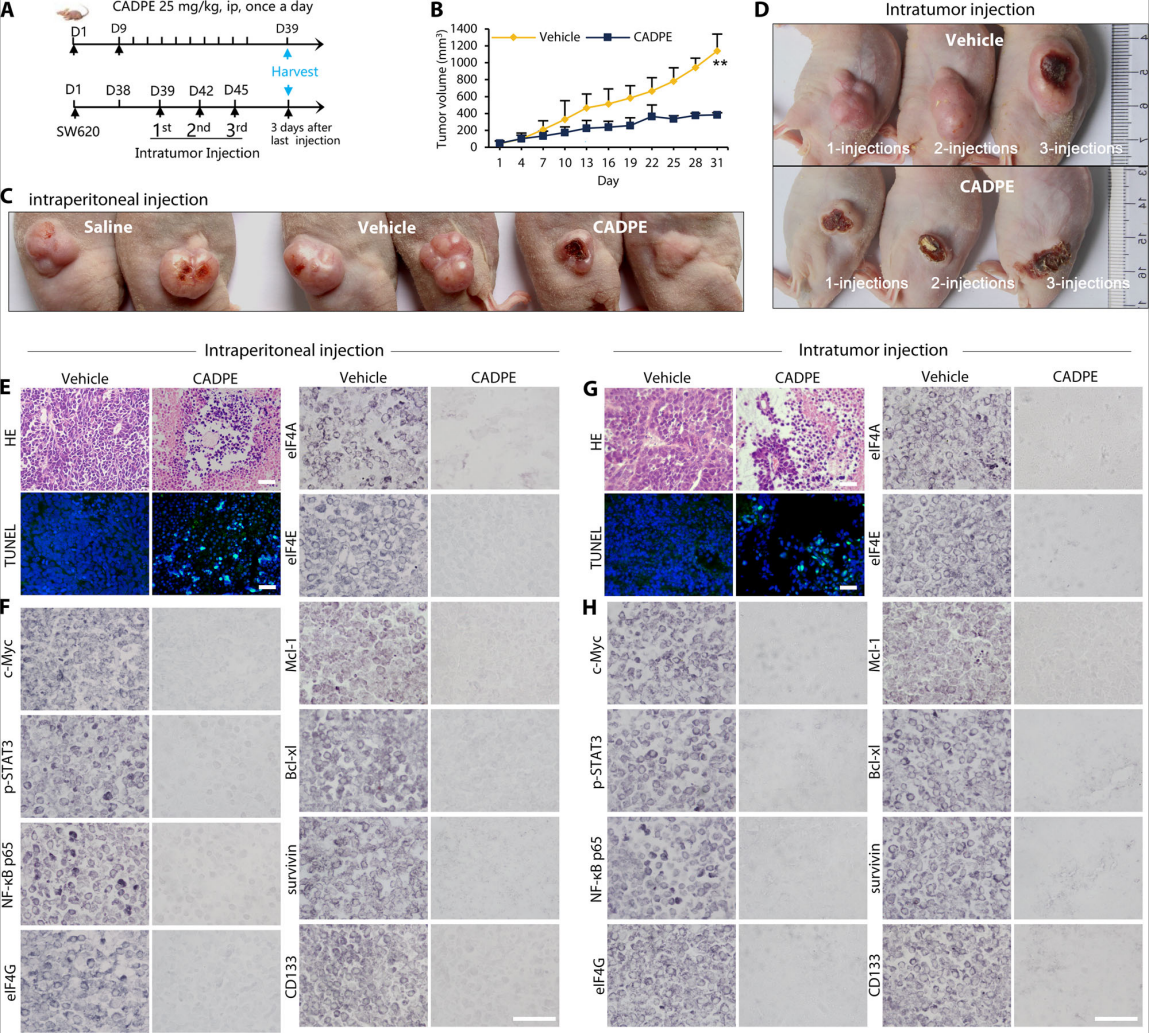
CADPE exerts potent in vivo anti-colorectal cancer effect through the same in vitro mechanism. **a** Schema depicts the in vivo experimental design. For intraperitoneal injection (i.p.), after the SW620 tumor reached about 100 mm^3^, mice were treated with saline, vehicle (CON), or CADPE (25 mg/kg, i.p. daily) for 31 days (*n* = 5). For intratumor injection (i.t.), after the tumor reached about 1000 mm^3^, mice were treated with saline, vehicle (CON), or CADPE (50 μM, i.t.) (*n* = 3). First injection: mice were only injected once at the first day and then the tumors were harvested after 15 days; second injection: mice were injected again after 3 days of the first injection and then the tumors were harvested after 12 days; third injection: mice were injected for the third time after 3 days of the second injection and then the tumors were harvested after 9 days. **b** CADPE significantly inhibited tumor growth in a SW620 BALB/c nude mice model (*n* = 5). **c**, **d** Representative images of SW620 xenografts in mice were shown. Animals were euthanized and photographed at the end time point of the treatment. **e**, **g** Hematoxylin and eosin (HE) staining and TUNEL assay. **e**–**h** Immunohistochemical (IHC) analysis of c-Myc, p-STAT3, NF-κB, eIF4G, eIF4A, eIF4E, Mcl-1, Bcl-xl, survivin, and CD133 of the residual tumor tissue. Bar: 30 μm. Data are presented as the mean ± SD, **p < 0.01 (vs. CON) by one-way ANOVA followed by Tukey’s test.

Finally, we tested whether CADPE would deplete the above key functional proteins that fuel residual disease in CRC tumor. The in vivo anticancer effects of CADPE were evaluated in SW620 xenograft nude mice through two different administration regimes (Fig. 7a). Through intraperitoneal injection, CADPE significantly suppressed tumor growth (Fig. 7b, c), without body weight alterations and any visible behavioral changes (data not shown), although CADPE was not able to exert its full antitumor efficacy because of its fast degradation by abundant mouse plasma carboxylesterase that is not present in human plasma^47^. Remarkably, through intratumor injection that can protect CADPE from the enzymolysis in mice plasma, CADPE induced complete tumor regression (Fig. 7d). Consistently, HE staining showed that the disrupted and disintegrated areas of tumor tissues in mice locally injected by CADPE are much larger than those of the tumor tissues in mice intraperitoneally injected by CADPE. However, abundant apoptotic cells identified by TUNEL assay were present in the collapsed tumor tissues regardless of the administration regimes (Fig. 7e, g). Moreover, the two administration routes potently decreased the abundance of pro-tumorigenic proteins, including c-Myc, p-STAT3, NF-κB, eIF4G, eIF4A, eIF4E, Mcl-1, Bcl-xl, survivin, and CD133 (Fig. 7e–h). These data indicated that CADPE exerted its potent in vivo anti-CRC efficacy through its similar in vitro mechanism.

## Discussion

This study demonstrates that CADPE not only kills heterogenetic CRC cells but also residual CRC cells. In terms of mechanism of action, CADPE inhibits oncogenic TFs c-Myc, NF-κB, and STAT3 and downregulates eIF4A, eIF4E, and eIF4G, leading to the decreased expression of oncogenic mRNAs, the impairment of Cap-dependent and Cap-independent oncogenic translations, and the consequent depletion of oncogenic signaling molecules and pro-survival factors including pro-residual disease molecules Bcl-xl, Mcl-1, survivin, CD133, and VEGF. A rational targeted polytherapy with an agent directed against the primary tumor driver plus a drug directed against a biological event that drives residual disease has been recently proposed^4^. Therefore, CADPE provides a novel and efficient strategy for simultaneously inhibiting these undruggable targets.

Currently, the molecule-inhibitor-binding strategy is used to suppress the dysregulated mRNA translation (such as targeting eIF4E with an antisense oligonucleotide), the eIF4E–cap interaction with the pronucleotide 4Ei-1, the eIF4E–eIF4G interaction with small molecules, and inactivating eIF4A with natural compounds. Importantly, CADPE dramatically downregulates the levels of eIF4E, eIF4G, and eIF4A, thus leading to the impairments of Cap-dependent translation and the eIF4G- or eIF4A-driven alterative translations that have been shown to cause cancer treatment failure^25,48^. Given that VEGF can be synthesized through Cap-dependent way and multiple alternative ways driven by the other *cis*-acting elements, including IRES, uORF, and G-quadruplex, resulting in occurrence of VEGF synthesis in diverse conditions including tumor-related stresses and drug treatments^25,48^, the depleted VEGF protein but not its mRNA abundance by CADPE serves as a typical result to verify the potent effectiveness of CADPE on Cap-dependent and alternative translations. This novel activity of CADPE enables it to deplete pro-tumorigenic proteins including ones required for maintaining residual cancer cells, and thereby to kill residual disease, which challenges both chemotherapies and targeted therapies for CRC. However, the suppressed translation is unable to explain why CADPE rapidly decreased c-Myc, p-STAT, and p-NF-κB levels, and whether protein degradation regulation involves in this fast effect of CADPE remains to be further investigated.

Moreover, although we did not focus on revealing how CADPE decreases the eIF4F levels and oncogenic mRNAs translation, our results indicate that, at an earlier time, CADPE can suppress the activity of the eIF4F complex through both initially reducing c-Myc level that drives increased protein synthesis through its roles dependent and independent of transcription^46^, and later downregulating more multiple signaling nodes such as STAT3, NF-κB, β-catenin, Notch1, mTOR, Raptor, and Rictor. This later action in turn limits the oncogenic mRNA levels that encode eIF4F and other pro-tumorigenic proteins and the eIF4F complex activity. To the best of our knowledge, CADPE is the first small molecule that impedes oncogenic translations through such multiple and cooperative mechanisms, resulting in an intensive and widespread downregulation of oncoproteins, including CD133 and VEGF that are highly resistant to the established anticancer therapeutic strategies.

## Supplementary information

Supplementary figure legends

Fig. S1

Fig. S2

Fig. S3

Fig. S4

Fig. S5

Fig. S6

Fig. S7

Fig. S8

Supplementary Tables
