# Supplementary material for: A small natural molecule CADPE kills residual colorectal cancer cells by inhibiting key transcription factors and translation initiation factors
Source: Cell Death Dis. 2020 Nov 15;11(11):982. doi: 10.1038/s41419-020-03191-5 (PMC7667164; doi:10.1038/s41419-020-03191-5)
Supplement: Supplementary file 1 — Supplementary figure legends [file 41419_2020_3191_MOESM1_ESM.docx]

**Supplementary figure and table legends**

**Fig. S1** CADPE induces apoptosis in CRC cells and senescence in HCT-8 cells. **a** Apoptosis rates of CRC cells were measured by flow cytometry after the treatment of CADPE (25 μM) for 48 and 72 h. **b** Senescence of HCT-8 cells was detected by using SA-β-gal staining after the treatment of CADPE (12.5 μM) for 48 h. Bar: 100 μm.

**Fig. S2** Effects of CADPE and regorafenib (Rego) on human normal colon fibroblast CCD-18Co cells. **a** CCD-18Co cells were treated with CADPE or regorafenib (Rego) using the indicated concentrations for 72 h and assayed by the SRB method. **b** CCD-18Co cells were treated with Rego (40 μM) or CADPE (1000 μM) for 48 h. Cell nuclei in blue were stained by DAPI and cytoskeleton in green by phalloidin. Bar: 50 μm. Data are presented as the mean ± SD (n = 5). **p*< 0.05, ***p*< 0.01, ****p*< 0.001 (vs. the untreated group) by one-way ANOVA followed by Tukey test.

**Fig. S3** Combination of Bcl-xl, Mcl-1, and survivin inhibitors induces apoptosis in CRC cells. **a** Apoptosis rates of CRC cells were measured by flow cytometry after the treatment of Bcl-xl inhibitor ABT737 (737, 0.5 μM and 3 μM), Mcl-1 inhibitor A-1210477 (477, 2 μM and 6 μM), survivin inhibitor YM155 (155, 3 μM and 1 μM), and a combination of two inhibitors or three inhibitors for 48 h, respectively. **b** Apoptosis rates of HCT-8 cells induced by 48 h treatment of CADPE (12.5 μM), 737 (3 μM), and their combination in HCT-8 cells.

**Fig. S4** CADPE and regorafenib (Rego) induce apoptosis in CRC cells. **a**, **b** Apoptosis rates of CRC cells were measured by flow cytometry after treating with CADPE (50, 100 μM) or Regorafenib (Rego, 10, 20 μM) for 72 h (n = 3). Data are presented as the mean ± SD ( n = 3), n.s *p*>0.05, **p*<0.05, ***p*<0.01 (vs. CADPE 50 μM), ^#^*p*<0.05, ^##^*p*<0.01, ^###^*p*<0.01 (vs. CADPE 100 μM) by two-tailed student′s *t*-test.

**Fig. S5** Stemness of the isolated colorectal cancer stem cells (CRCSCs) and effects of CADPE and regorafenib (Rego) on cell viability of HCT-15, HCT-116, and HCT-8 cancer stem cells (CSCs). **a** Relative mRNA levels of the stemness makers CD44, CD133, and Notch-1 in HCT-116 parental cells and HCT-116 CSCs were determined by qRT-PCR. Data are presented as the mean ± SD (n = 3), ***p*< 0.01 by two tailed student′s *t*-test. **b** Protein levels of the stemness makers CD44, CD133, and Notch-1 in HCT-116 parental cells and HCT-116 CSCs were analyzed by western blot. **c** Effect of CADPE and Rego on cell viability of HCT-8, HCT-15 and HCT-116 CSCs isolated and enriched from spheres. CRCSCs were cultured with CADPE (10, 25, 50, 100 μM) or Rego (1, 5, 10, 30 μM) for 72 h in medium contained 10% FBS. Data are presented as the mean ± SD (n = 5), ***p*< 0.01, ****p*< 0.001 (vs. the untreated group) by one-way ANOVA followed by Tukey test.

**Fig. S6** Effects of CADPE and regorafenib (Rego) on colorectal cancer stem cells (CRCSCs). **a**, **b** HCT-15 and HCT-116 cancer stem cells (CSCs) were treated with CADPE (25 μM) or regorafenib (Rego, 5 μM) for 8 days in cancer stem cell medium. After 8 days, the drugs were removed and the cells were cultured in fresh cancer stem cell medium for another 7 days. Representative images are shown and sphere growth was scored by counting the number of spheres possessing >50 μm. **c** HCT-15 and HCT-116 CSCs were allowed to form spheres in suspension with cancer stem cell medium for 7 days before the treatment of 72 h with CADPE (50 μM) or Rego (10 μM) and CADPE collapsed the spheres of CRCSCs. **d** HCT-15 and HCT-116 CSCs were treated with CADPE (25, 50, 100 μM) for 72 h and CADPE significantly induced apoptosis in HCT-15 and HCT-116 CSCs. Data are presented as the mean ± SD (n = 3), **p*< 0.05, ****p*< 0.001 (vs. the untreated group) by one-way ANOVA followed by Tukey test.

**Fig. S7** CADPE downregulates stemness makers, anti-apoptotic proteins, and key oncogenic transcriptional factors in HCT-15 and HCT-116 stem cells (CSCs) after the treatment of CADPE at 100 μM for 24 h. **a** Effect of CADPE on the levels of stemness makers CD44, CD133, Bmi-1, and Notch-1. **b** Effect of CADPE on the levels of anti-apoptotic proteins Bcl-xl, Mcl-1, survivin. **c** Effect of CADPE on the levels of key oncogenic transcriptional factors p-STAT3, STAT3, and c-Myc.

**Fig. S8** Effect of inhibition of multiple oncogenic transcription factors and protein translation on apoptosis in CRC cells. Apoptosis rates of SW620, HCT-15, and HCT-8 cells were measured by flow cytometry after treating with c-Myc inhibitor (10058-F4, F4, 30 μM), STAT3 inhibitor (Ruxolitinib, Rux, 20 μM), NF-κB inhibitor (Bay 11-7085, Bay, 20 μM), competitive eIF4E/eIF4G interaction inhibitor (4EGI-1, 4EGI, 10 μM), or their random combinations (n = 3).

Table S1_._ Lists of antibodies used for the western blot

Table S2. Primers used in quantitative real-time polymerase chain reaction (qRT-PCR)

Table S3. Activity of regorafenib (Rego) and CADPE in inhibiting the proliferation of colorectal cancer cells (IC_50_: μM)
