# Supplementary figures and images for: A small natural molecule CADPE kills residual colorectal cancer cells by inhibiting key transcription factors and translation initiation factors
Source: Cell Death Dis. 2020 Nov 15;11(11):982. doi: 10.1038/s41419-020-03191-5 (PMC7667164; doi:10.1038/s41419-020-03191-5)

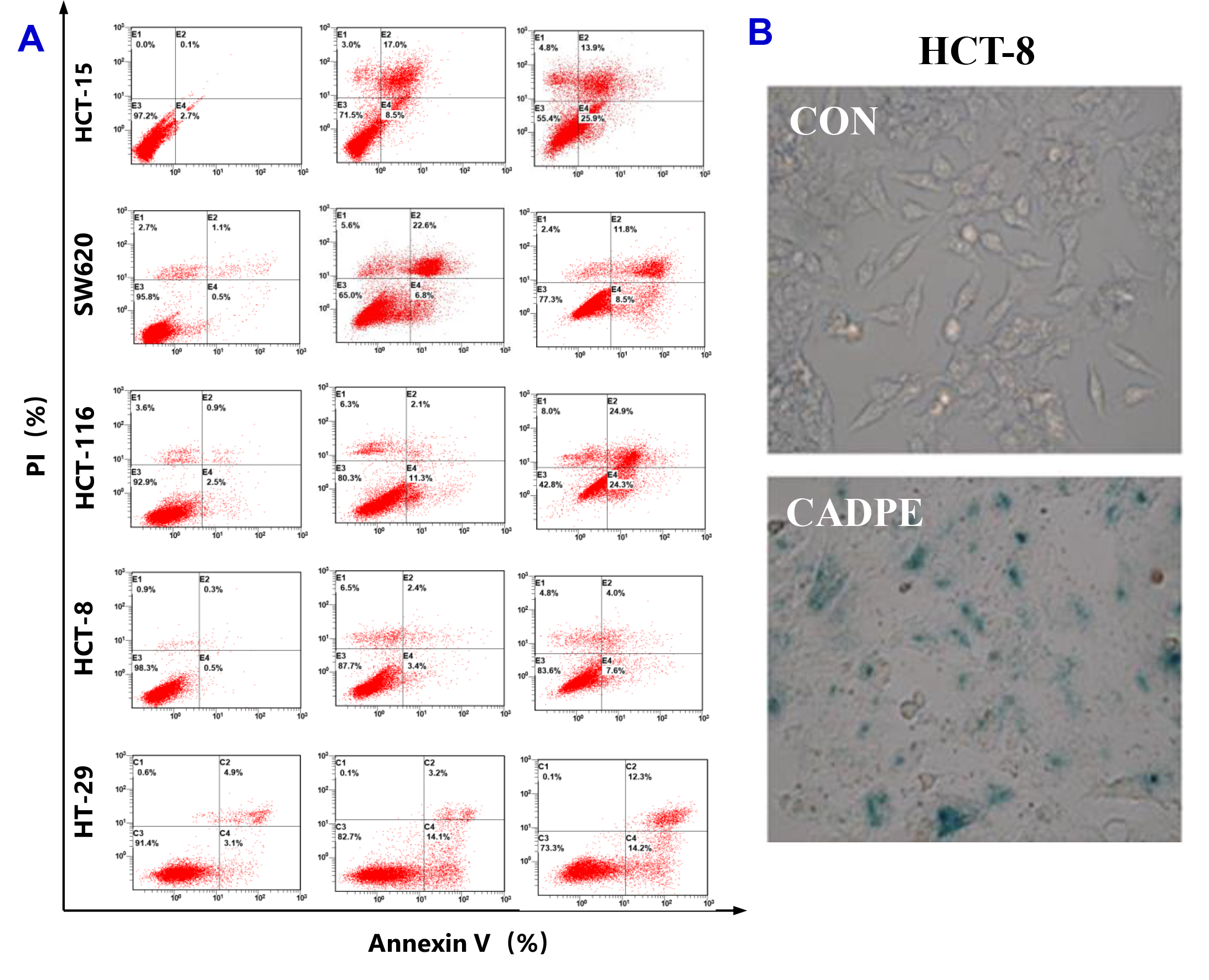

Supplement: Supplementary file 2 — Fig. S1 [file 41419_2020_3191_MOESM2_ESM.tif]

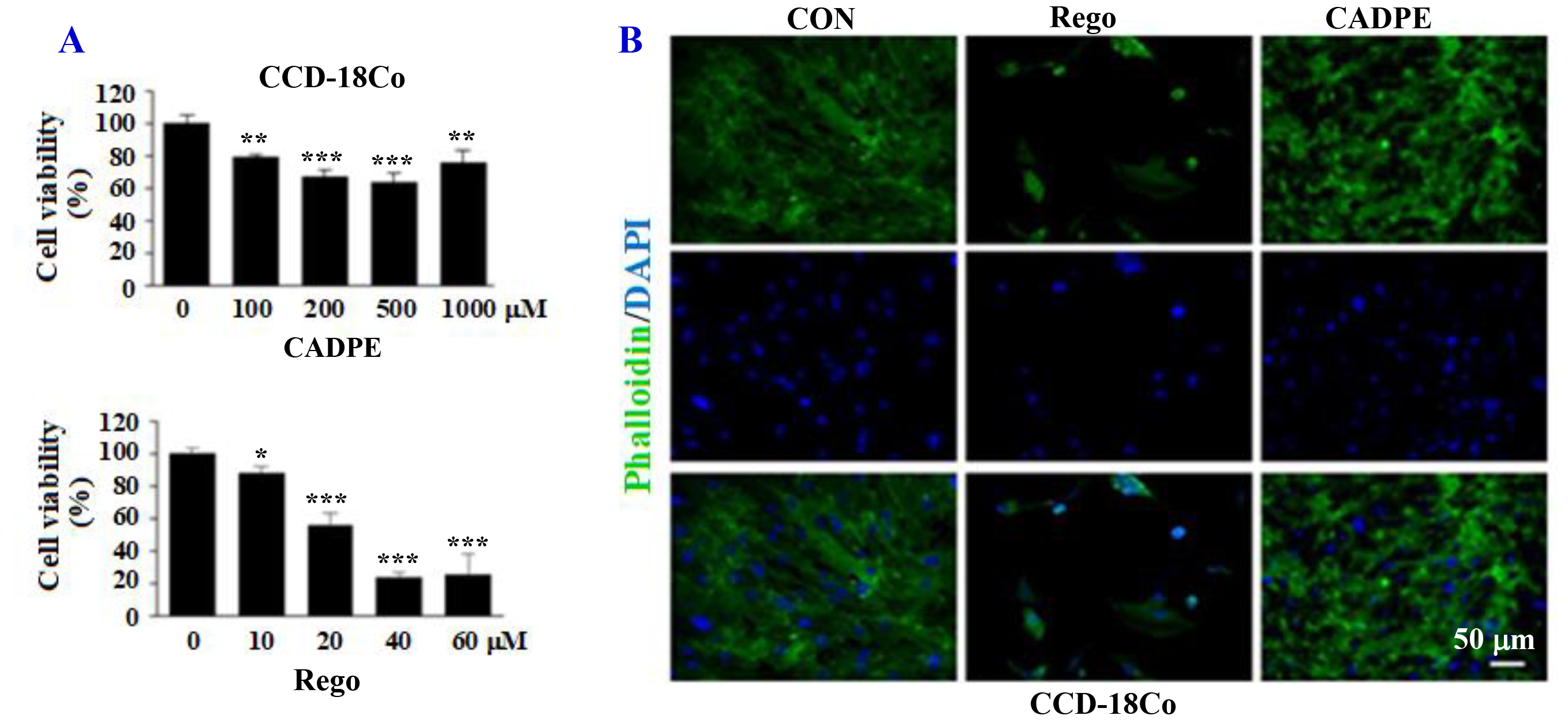

Supplement: Supplementary file 3 — Fig. S2 [file 41419_2020_3191_MOESM3_ESM.tif]

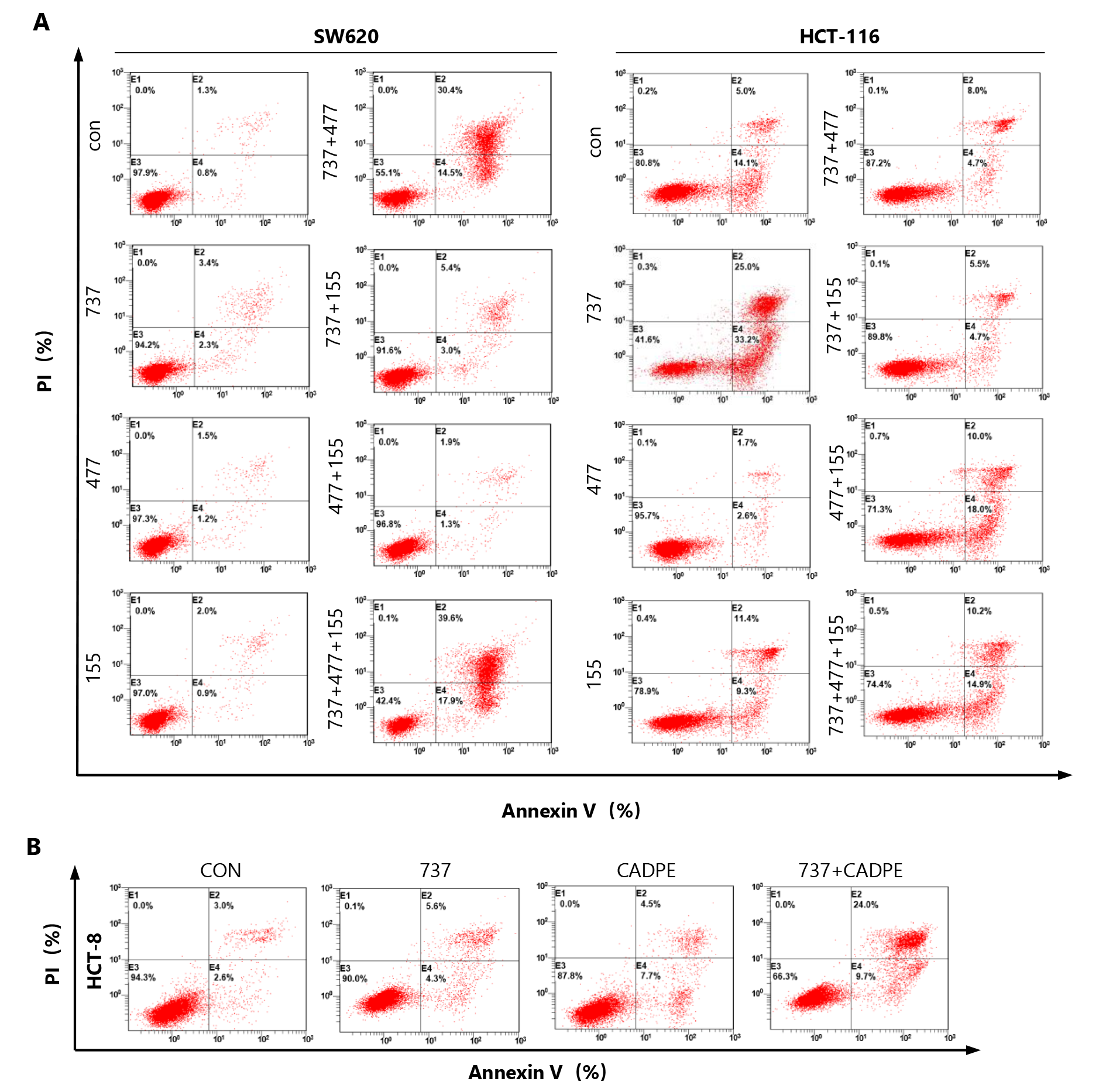

Supplement: Supplementary file 4 — Fig. S3 [file 41419_2020_3191_MOESM4_ESM.tif]

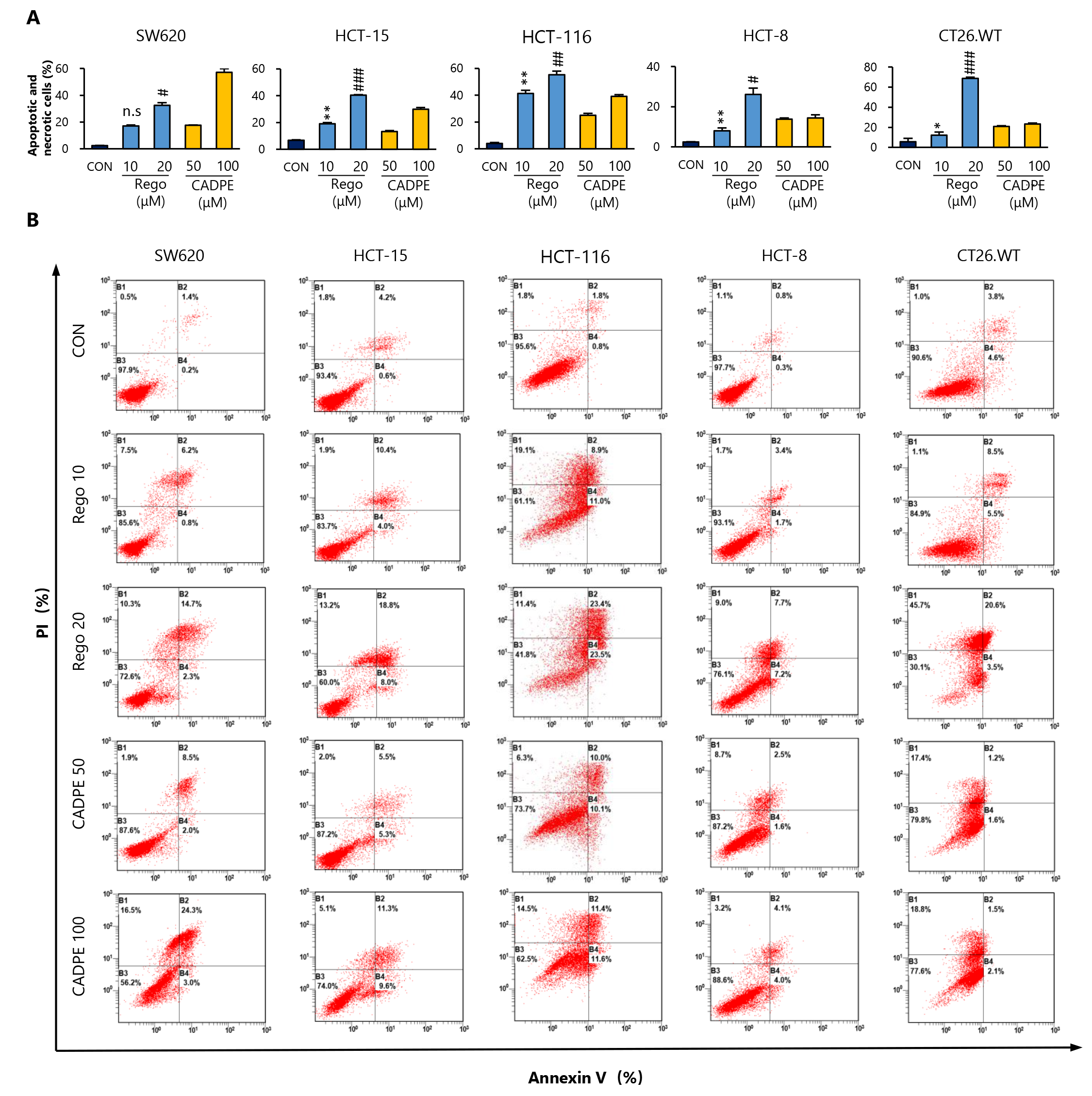

Supplement: Supplementary file 5 — Fig. S4 [file 41419_2020_3191_MOESM5_ESM.tif]

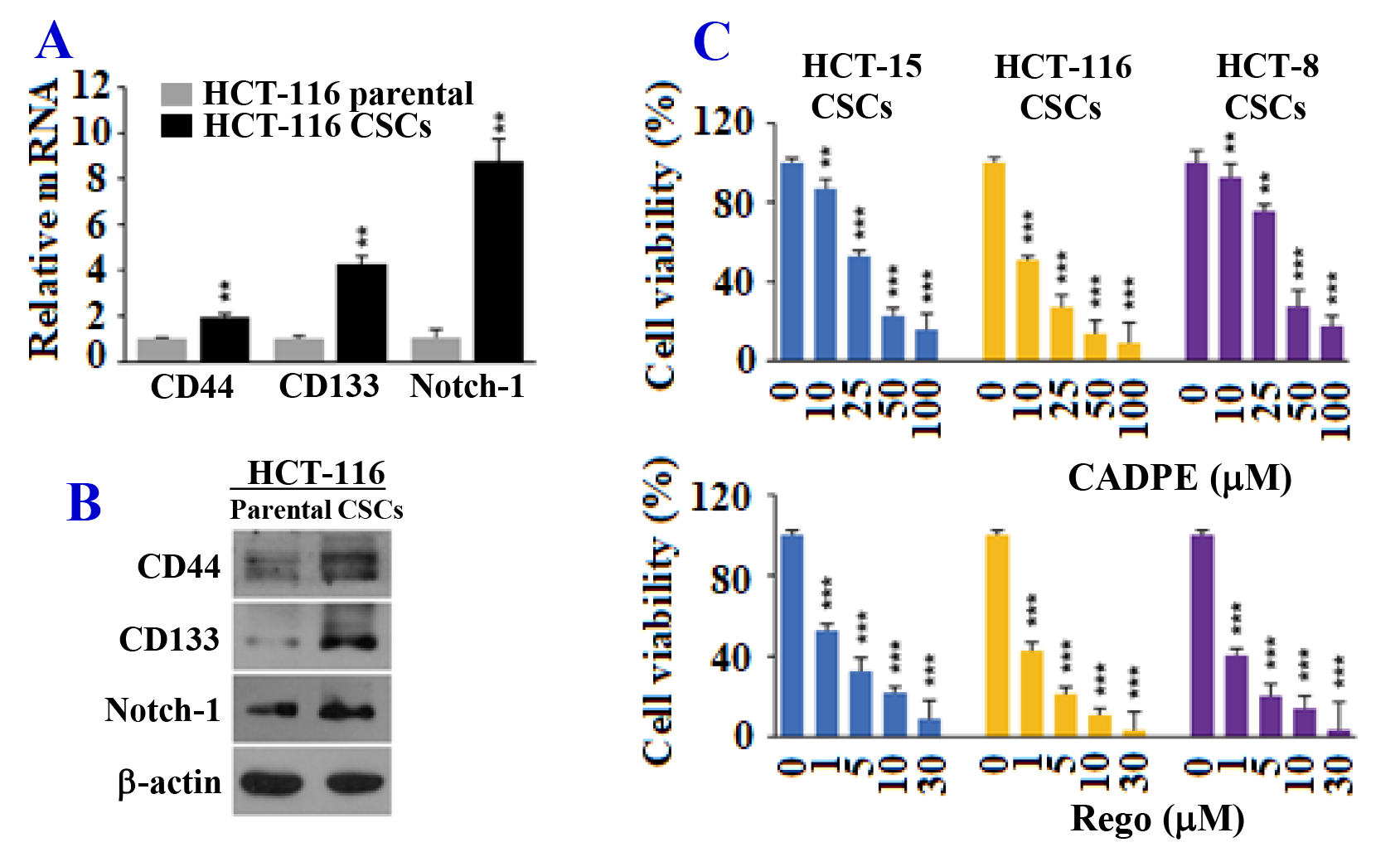

Supplement: Supplementary file 6 — Fig. S5 [file 41419_2020_3191_MOESM6_ESM.tif]

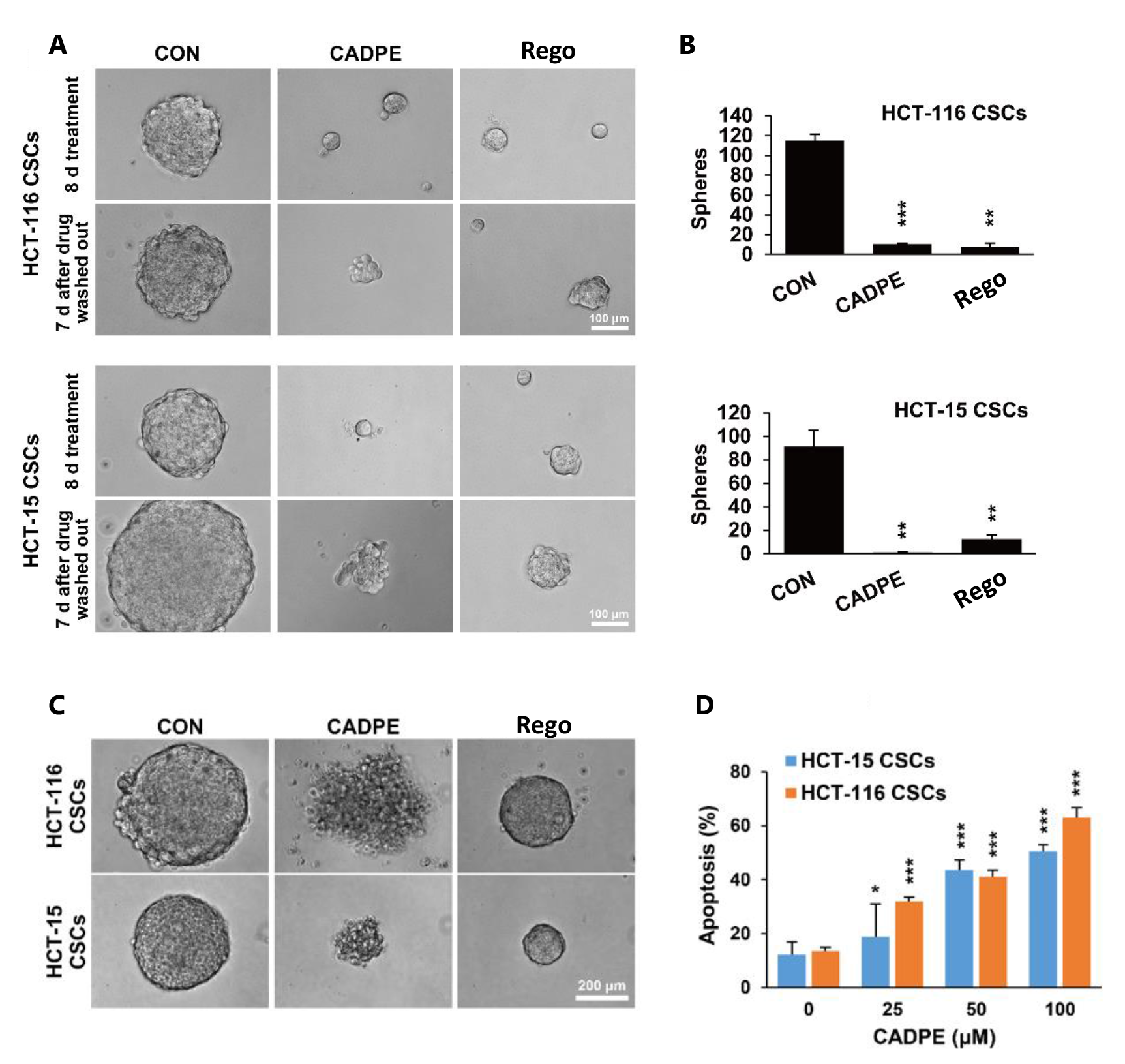

Supplement: Supplementary file 7 — Fig. S6 [file 41419_2020_3191_MOESM7_ESM.tif]

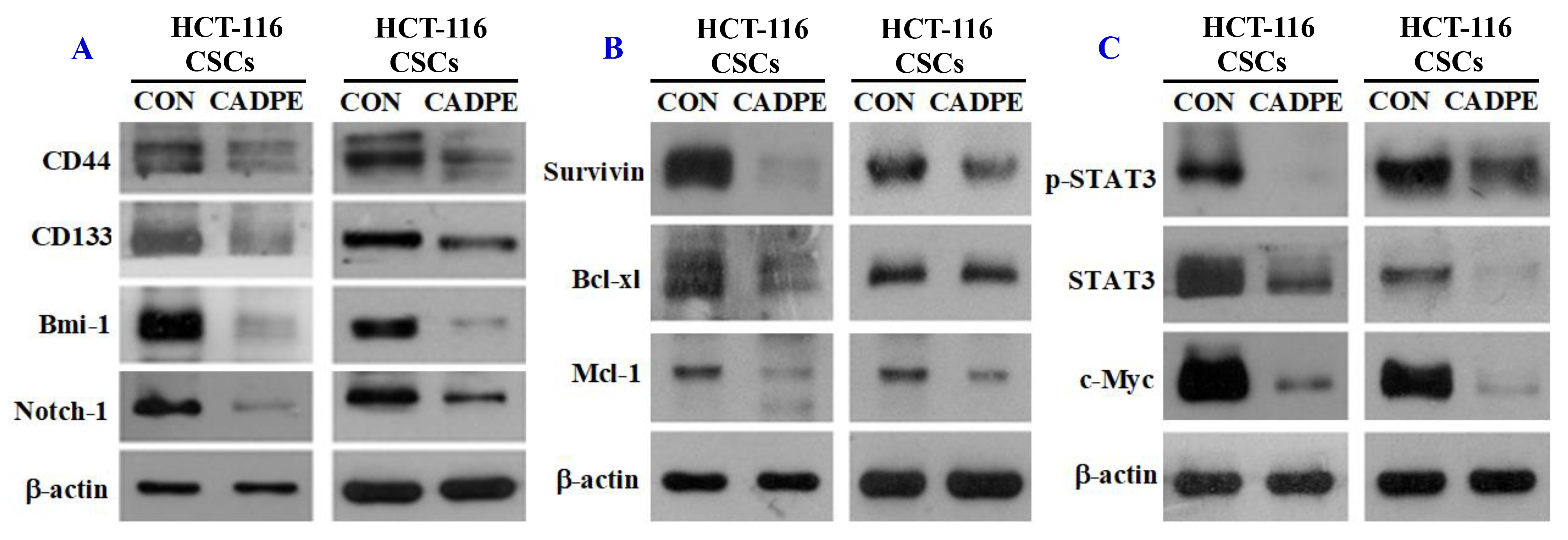

Supplement: Supplementary file 8 — Fig. S7 [file 41419_2020_3191_MOESM8_ESM.tif]

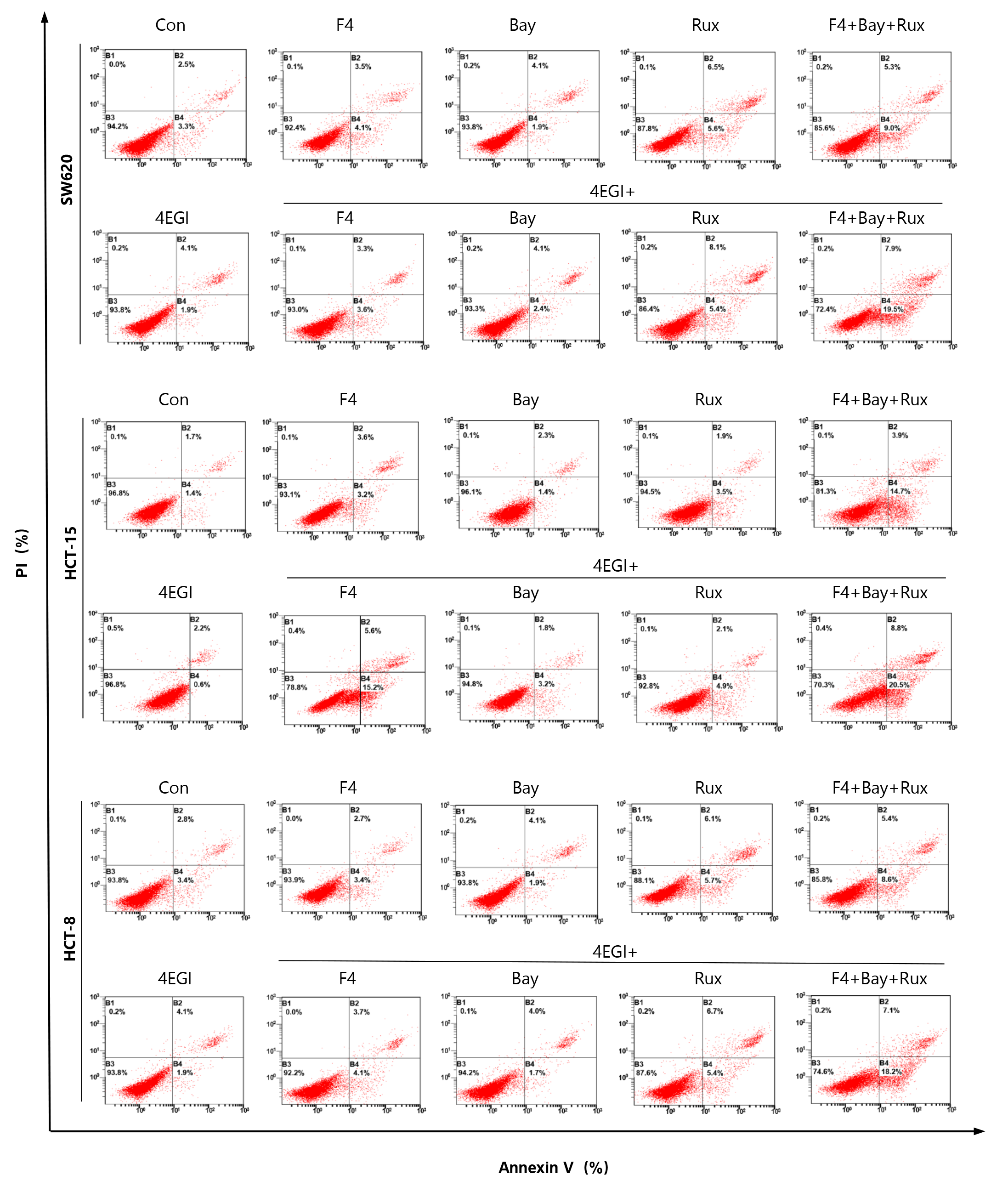

Supplement: Supplementary file 9 — Fig. S8 [file 41419_2020_3191_MOESM9_ESM.tif]
