# Supplementary material for: A small natural molecule CADPE kills residual colorectal cancer cells by inhibiting key transcription factors and translation initiation factors
Source: Cell Death Dis. 2020 Nov 15;11(11):982. doi: 10.1038/s41419-020-03191-5 (PMC7667164; doi:10.1038/s41419-020-03191-5)
Supplement: Supplementary file 10 — Supplementary Tables [file 41419_2020_3191_MOESM10_ESM.docx]

Table S1_._ Lists of antibodies used for the western blot

| **Antibody** | **Species** | **Producer** | **Cat. No.** | **Isotype** | **Clonality** | **RRID** | **Dilution (WB)** | **Dilution (IHC)** |
| --- | --- | --- | --- | --- | --- | --- | --- | --- |
| c-Myc | Rabbit | Abcam | ab32072 | IgG | Monoclonal | AB_731658 | 1:5000 | 1:100 |
| p-STAT3 Y705 | Rabbit | CST | #9145 | IgG | Monoclonal | AB_2491009 | 1:2000 | 1:100 |
| STAT3 | Rabbit | HuaBio | ET1067-38 | IgG | Polyclonal | / | 1:2000 |  |
| p-NF-κB S536 | Rabbit | CST | #3033 | IgG | Monoclonal | AB_331284 | 1:2000 | 1:100 |
| NF-κB | Rabbit | HuaBio | ER0815 | IgG | Polyclonal | / | 1:3000 |  |
| p-Akt T308 | Rabbit | CST | #13038 | IgG | Monoclonal | AB_2629447 | 1:2000 |  |
| p-Akt S473 | Rabbit | CST | #4060 | IgG | Monoclonal | AB_2315049 | 1:2000 |  |
| Akt | Rabbit | Ruiying Biological | RLM3461 | IgG | polyclonal | / | 1:2000 |  |
| p-mTOR S2448 | Rabbit | CST | #5536 | IgG | Monoclonal | AB_10691552 | 1:3000 |  |
| mTOR | Rabbit | HuaBio | ET1608-5 | IgG | Monoclonal | / | 1:3000 |  |
| p-Rictor T1135 | Rabbit | CST | #3806 | IgG | Monoclonal | AB_10557237 | 1:3000 |  |
| Rictor | Rabbit | Proteintech | 27248-1-AP | IgG | polyclonal | AB_2179839 | 1:3000 |  |
| Raptor | Rabbit | Proteintech | 20984-1-AP | IgG | polyclonal | AB_11182390 | 1:3000 |  |
| p-p70S6K T389 | Rabbit | CST | #9234 | IgG | Monoclonal | AB_2269803 | 1:2000 |  |
| p70 | Rabbit | CST | #2708 | IgG | Monoclonal | / | 1:2000 |  |
| p-4EBP1S65 | Rabbit | CST | #9451 | IgG | Polyclonal | AB_2269803 | 1:3000 |  |
| 4EBP1 | Mouse | Proteintech | 60246-1-Ig | IgG2b | Monoclonal | AB_2097832 | 1:2000 |  |
| eIF4E | Rabbit | Abcam | ab33766 | IgG | Monoclonal | AB_732125 | 1:200 | 1:50 |
| eIF4A | Rabbit | CST | #2013 | IgG | Monoclonal | AB_2097832 | 1:200 | 1:50 |
| eIF4G | Rabbit | CST | #2469 | IgG | Monoclonal | AB_2096028 | 1:200 | 1:50 |
| Bcl-xl | Rabbit | Epitomics | 1018-1 | IgG | Polyclonal | / | 1:5000 | 1:200 |
| Mcl-1 | Rabbit | HuaBio | ET1606-14 | IgG | Polyclonal | / | 1:2000 | 1:100 |
| Bax | Rabbit | Epitomics | 1063-1 | IgG | Polyclonal | / | 1:3000 |  |
| BIM-1 | Rabbit | Santa cruz | sc-11425 | IgG | Polyclonal | / | 1:1000 |  |
| Survivin | Rabbit | CST | #2808 | IgG | Monoclonal | AB_2063948 | 1:2000 | 1:100 |
| VEGF | Rabbit | Antibodies-Online | ABIN238296 | IgG | Polyclonal | AB_10777892 | 1:1000 |  |
| CD44 | Rabbit | Abcam | ab189524 | IgG | Monoclonal | / | 1:5000 |  |
| CD133 | Rabbit | Abcam | ab226355 | IgG | Monoclonal | / | 1:1000 | 1:100 |
| Notch1 | Rabbit | US Biological | N5375-05 | IgG | Polyclonal | AB_2251467 | 1:1000 |  |
| β-actin | Mouse | Abcam | ab8226 | IgG1 | Monoclonal | AB_306371 | 1:20000 |  |
| HRP-labeled Goat  Anti-Rabbit IgG(H+L) | Goat | Beyotime Biotechnology | A0208 | IgG | Polyclonal | / | 1:2500 |  |
| HRP-labeled Goat  Anti-Mouse IgG(H+L) | Goat | Beyotime Biotechnology | A0216 | IgG | Polyclonal | / | 1:2500 |  |
| Alexa Fluor  488  labeled  Goat  Anti-Rabbit IgG(H+L) | Goat | Yeasen Biotech Co., Ltd. | 34206ES60 | IgG | Polyclonal | / | 1:2000 |  |

Table S2. Primers used in quantitative real-time polymerase chain reaction (qRT-PCR)

| Genes | Primers | Sequence |
| --- | --- | --- |
| BCL-XL | F | ATTGGTGAGTCGGATCGCAGC |
|  | R | AGAGAAGGGGGTGGGAGGGTA |
| MCL-1 | F | CATTCCTGATGCCACCTTCT |
|  | R | TCGTAAGGACAAAACGGGAC |
| BIRC5 (Survivin) | F | TGAACTTCAGGTGGATGAGGAGA |
|  | R | GTCTAATCACACAGCAGTGGCAA |
| ACTA1 | F | TCACCCACACTGTGCCCATCTACGA |
|  | R | CAGCGGAACCGCTCATTGCCAATGG |
| MYC (c-Myc) | F | ATGTCCTGAGCAATCACCTATG |
|  | R | AAGTTCTTTTATGCCCAAAGTCC |
| NF-κB | F | GGTCCGCTGAAAGGACTCTT |
|  | R | GAATTCCAGTACCTGCCAGA |
| STAT3 | F | CCTGCTAAAATCAGGGGTCC |
|  | R | GTCTCTCCCCCTCGGCT |
| MTOR | F | TCCGGCTGCTGTAGCTTATT |
|  | R | TGAGAGAGCTGCCAAGTGC |
| RAPTOR | F | GATCGATCCAGCATTCCAAG |
|  | R | TCAGAGCTGGAGGATGAAGG |
| RICTOR | F | ATCTGGCCACATTTTGGAGA |
|  | R | AGAACCTCCGAGTACGAGGG |
| EIF4E | F | AGGAGGTTGCTAACCCAGAAC |
|  | R | CATCTTCCCACATAGGCTCAA |
| EIF4A | F | TGCTTAACCGGAGATACCTGTC |
|  | R | GTCCCTCATGAACTTCTTGGTC |
| EIF4G | F | CCCAACTGTAGAAGGCATCC |
|  | R | CTCCAGGCCCTTGTAGTGAC |
| VEGF | F | TTGCCTTGCTGCTCTACCTCCA |
|  | R | GATGGCAGTAGCTGCGCTGATA |
| CD44 | F | GACAAGTTTTGGTGGCACG |
|  | R | CACGTGGAATACACCTGCAA |
| CD133 | F | AAGCATTGGCATCTTCTATGG |
|  | R | AAGCACAGAGGGTCATTGAGA |
| Bmi-1 | F | CAAGAGTTGCGGCGTATTGGAG |
|  | R | ACACCAGGCGGACAATGTAACG |
| Notch1 | F | TCAATGTTCGAGGACCAGATG |
|  | R | TCACTGTTGCCTGTCTCAAG |

Table S3. Activity of regorafenib (Rego) and CADPE in inhibiting the proliferation of colorectal cancer cells (IC_50_: μM)

| Cancer cells | SW620 | HCT-15 | HCT-116 | HCT-8 | HT-29 |
| --- | --- | --- | --- | --- | --- |
| Rego | 5.93 ± 0.35 | 2.81 ± 0.50 | 4.12 ± 0.52 | 2.14 ± 0.17 | 6.99 ± 0.72 |
| CADPE | 7.74 ± 0.18 | 7.47 ± 1.73 | 12.16 ± 0.53 | 18.07 ± 2.24 | 32.30 ± 1.89 |
